# CAR-T cells targeting CD155 reduce tumor burden in preclinical models of leukemia and solid tumors

**DOI:** 10.1172/JCI189920

**Published:** 2025-06-06

**Authors:** Tianchen Xiong, Ge Wang, Peng Yu, Zhenlong Li, Debao Li, Jianying Zhang, Song Lu, Ruiqi Yang, Xiaolong Lian, Jianhong Mi, Rui Ma, Zhiyao Li, Guido Marcucci, Tingting Zhao, Michael A. Caligiuri, Jianhua Yu

**Affiliations:** 1 Department of Hematology & Hematopoietic Cell Transplantation and; 2 Hematologic Malignancies Research Institute, City of Hope National Medical Center, Los Angeles, California, USA.; 3 Department of Neurology, Xiangya Hospital, Central South University, Changsha, China.; 4 Chongqing International Institute for Immunology, Chongqing, China.; 5 Department of Computational and Quantitative Medicine and; 6 Gehr Family Center for Leukemia Research, Hematologic Malignancies Research Institute, City of Hope National Medical Center, Los Angeles, California, USA.; 7 Division of Hematology & Oncology, Department of Medicine, School of Medicine,; 8 Institute for Precision Cancer Therapeutics and Immuno-Oncology, Chao Family Comprehensive Cancer Center, and; 9 The Clemons Family Center for Transformative Cancer Research, University of California, Irvine, California, USA.

**Keywords:** Oncology, Therapeutics, Cancer immunotherapy

## Abstract

CAR-T cells are a powerful yet expensive tool in cancer immunotherapy. Although their use in targeting hematological malignancies is well established, using a single CAR-T cell therapy to treat both hematological and solid tumors, which can reduce cost, remains largely unexplored. In this study, we identified CD155, an adhesion molecule that is upregulated during tumor progression, as a target for CAR-T cell therapy in both leukemia and solid tumors. We engineered CAR-T cells using human and mouse anti–CD155 antibodies generated from a Berkeley Lights’ Beacon platform. These CAR-T cells demonstrated potent antitumor activity, significantly reducing tumor burden in preclinical models of acute myeloid leukemia, non–small cell lung cancer, and pancreatic cancer. To reduce potential allogeneic rejection, we generated CAR-T cells using humanized anti–CD155 antibody sequences that retained efficacy. Additionally, murine CAR-T cells targeting mouse CD155 exhibited limited toxic side effects in immunocompetent mice, highlighting the favorable safety profile of this therapy. These findings suggest that CD155 can be targeted by CD155 CAR-T cells safely and effectively, representing an innovative cellular therapeutic strategy that has the potential to expand its scope across both AML and multiple solid tumors, thereby lowering the cost of cellular immunotherapy, especially as allogenic, universal, and off-the-shelf CAR-T cell therapies advance to the clinic.

## Abstract 2

CD155-targeted CAR-T cells show strong anti-tumor effects in both leukemia and solid tumors, with limited toxic side effects and broad therapeutic potential.

## Introduction

Chimeric antigen receptor T-cell (CAR-T) immunotherapy has revolutionized the treatment of B-cell malignancies, achieving significant success in treating lymphoid leukemias, lymphomas, and myelomas (1–4). However, extending this success to acute myeloid leukemia (AML) and solid tumors has proven challenging, with outcomes thus far eluding success (5–9). Moreover, current CAR-T cells are costly. Discovering a target that can be the focus of CAR-T cells for treating both AML and solid tumors not only could be convenient in clinical practice but could also reduce costs, especially as allogenic, universal and off-the-shelf CAR-T cells are being developed. This highlights the need for new target identification and novel designs for both CARs and their effector cells to expand the therapeutic potential of CAR-T cell therapy (10).

CD155, also known as the poliovirus receptor (PVR), is an adhesion molecule involved in cell adhesion, migration, and proliferation (11, 12). Although its expression is absent or modest in most normal tissues, CD155 is frequently upregulated in a variety of tumor types, including colon, breast, pancreatic, and lung cancers, as well as hematologic malignancies such as AML (13–17). Increased CD155 expression enhanced tumor cell invasion, migration, and proliferation, whereas reducing CD155 expression significantly diminished tumor growth and metastatic spread, in various preclinical models (11, 18–20). Furthermore, the high expression of CD155 is associated with tumor progression and poor prognosis (21).

Beyond its protumorigenic functions (11, 18–20), CD155 also plays an immunoregulatory role in tumor progression. It interacts with the activating receptor DNAX-associated molecule-1 (DNAM-1 [or CD226]), primarily expressed on T cells, NK cells, B cells, and monocytes, promoting early tumor recognition and elimination (22, 23). However, in advanced stages of cancer, inhibitory receptors like TIGIT and CD96, which are upregulated on NK and T cells, outcompete DNAM-1 for CD155 binding (24–26). These inhibitory receptors bind CD155 with higher affinity, suppressing DNAM-1–mediated activation and impairing NK and T cell cytotoxicity (24, 27–30). Additionally, chronic exposure to CD155 leads to the downregulation of DNAM-1, further dampening the immune response (31, 32).

Given its aberrant expression in tumors and the critical role of the CD155/DNAM-1/TIGIT axis in tumor progression and immune regulation, targeting CD155 has emerged as a promising therapeutic strategy (21, 33–35). Several approaches that leverage CD155 interactions are under investigation, including recombinant oncolytic polioviruses (e.g., PVSRIPO) that selectively target tumor cells expressing CD155 (36, 37), checkpoint blockade therapies that inhibit receptors like TIGIT to restore immune function (38–40), and DNAM-1–based adoptive cell therapies designed to enhance immune cytotoxicity against CD155-expressing tumors (41, 42). In addition, recent studies have demonstrated the therapeutic efficacy of anti–CD155 blocking antibodies in preclinical tumor models, either as monotherapy or in combination with other immunotherapies such as CD33 CAR-T cells or PD-1 blockade, further supporting CD155 as a viable immunotherapeutic target (43, 44). Despite these advances, direct targeting of CD155 using modalities like CD155-specific CAR-T cells remains largely underexplored.

In this study, we developed a CAR-T cell therapy targeting CD155 for the treatment of both AML and solid tumors and evaluated its therapeutic efficacy and safety in preclinical models. CD155 CAR-T cells demonstrated potent antitumor activity in both AML and solid tumors in vitro and in vivo. Additionally, safety assessments in an immunocompetent mouse model revealed limited toxicity, suggesting a favorable safety profile. CD155-targeted CAR-T cells, therefore, may represent a potential approach for treating a wide range of cancers, including both AML and solid tumors, potentially expanding the scope of and reducing costs of effective cellular immunotherapy of cancer.

## Results

### CD155 is expressed on AML cell lines and primary AML samples.

**Figure 1:**
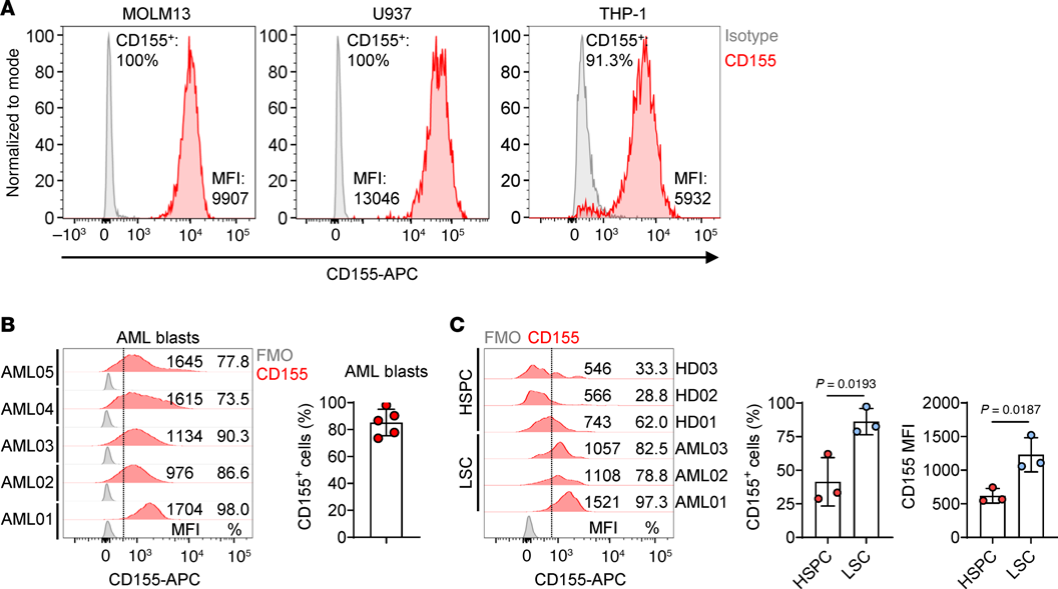
CD155 is expressed on AML cell lines and primary AML samples. (**A**) Representative histograms showing the expression of CD155 on AML cell lines. (**B**) Representative histograms (left) and statistics (right) of the percentage showing the expression of CD155 on primary AML blasts (*n* = 5 individual donors). (**C**) Representative histograms (left) and statistics of the percentage (middle) and MFI (right) showing the expression of CD155 on HSPCs from healthy donors (*n* = 3 individual donors) and on CD34^+^CD38^-^ LSCs from patients with AML (*n* = 3 individual donors). Data represent the mean ± SD (**B** and **C**) and were analyzed by 2-tailed Student’s *t* tests (**C**).

CD155 is upregulated during tumor progression (13–17). To assess the potential of CD155 as a target for CAR-T cell therapy in AML, we first evaluated its surface expression on 3 AML cell lines (MOLM13, U937, and THP-1) as well as on primary AML blasts from patients with AML. Despite some variation between samples, all 3 cell lines and blasts from 5 patients with AML had moderate to high levels of CD155 expression (Figure 1, A and B). Notably, greater than 70% of leukemia stem cells (LSCs) from the patients with AML tested were positive for CD155 (Figure 1C). Although CD155 expression was also detected in a subset of hematopoietic stem and progenitor cells (HSPCs) from healthy donors, its level, both in terms of the percentage of positive cells and MFI, was significantly lower than the levels in LSCs from patients with AML (Figure 1C). These findings collectively suggest CD155 could be an effective target for CAR-T cell therapy of AML.

### Phenotypic and functional assessment of CD155 CAR-T cells.

Following the development of our mouse anti–human antibody against CD155 and the retroviral transduction of our CD155-CAR construct into human T cells (see Methods), we next analyzed the phenotype of CD155 CAR-T cells 10 days after CAR transduction. CD155 CAR-T cells showed an increased proportion of CD4^+^ T cells and a decreased proportion of CD8^+^ T cells compared with mock T cells (Supplemental Figure 1A; supplemental material available online with this article; https://doi.org/10.1172/JCI189920DS1). In addition, flow cytometry revealed enhanced proliferation of CD155 CAR-T cells, indicated by a reduced MFI of CellTrace Violet fluorescence compared with mock T cells (Supplemental Figure 1B). Cell death rates between CD155 CAR-T cells and mock T cells were not significantly different (Supplemental Figure 1C). Previous studies have shown that CD4^+^ CAR-T cells, particularly those with a 4-1BB costimulatory domain, tend to expand more than CD8^+^ CAR-T cells (45). Consistent with this, we observed greater proliferation of CD4^+^ CAR-T cells over CD8^+^ CAR-T cells in our system (Supplemental Figure 1D), which may explain the higher proportion of CD4^+^ CAR-T cells.

To further understand the functional state of CD155 CAR-T cells, we assessed their activation status by evaluating the expression of CD69 and CD25, both well-established markers of T cell activation (46, 47). Our results showed that CD155 CAR-T cells had a significantly higher proportion of CD69^+^ and CD25^+^ cells in both CD4^+^ and CD8^+^ subsets compared with mock T cells (Supplemental Figure 1E), indicating enhanced activation of CD155 CAR-T cells. Next, we assessed the memory differentiation profile of CD155 CAR-T cells in both CD4^+^ and CD8^+^ populations. In CD4^+^ CAR-T cells, we observed a marked increase in the proportion of effector memory T cells alongside a notable reduction in central memory T cells, compared with mock T cells. In contrast, no significant differences were found across memory subsets within the CD8^+^ CAR-T cell population (Supplemental Figure 1F), demonstrating that CD155 CAR-T cells selectively influence memory differentiation in CD4^+^ T cells. Collectively, our data indicate that CD155 CAR-T cells exhibit enhanced proliferation, activation, and a distinct memory profile, particularly within the CD4^+^ subset. This suggests that CD155 CAR-T cells are primed for rapid and effective antitumor responses, highlighting their therapeutic potential.

### CD155 CAR-T cells are functional and effectively eliminate AML cell lines in vitro.

**Figure 2:**
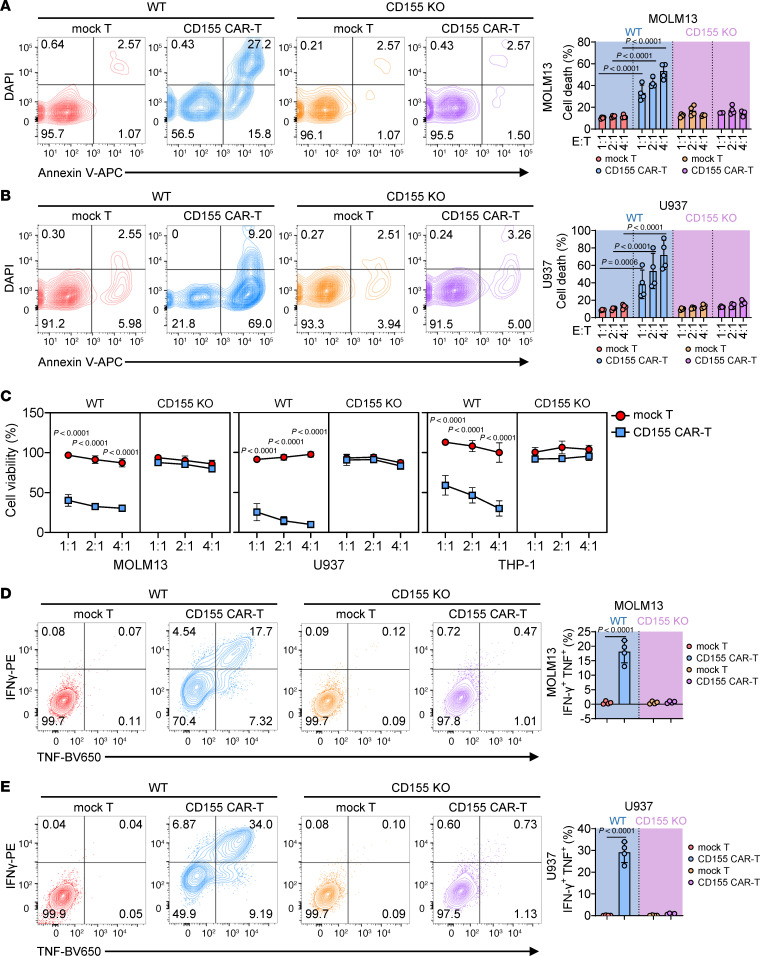
CD155 CAR-T cells are functional and effectively eliminate AML cell lines in vitro. (**A**) Representative flow cytometry plots (left) and statistics (right) showing the percentage of wild-type (WT) or CD155-KO MOLM13 cell death cocultured at indicated ratios with mock T or CD155 CAR-T cells for 4 hours (*n* = 4 individual donors). (**B**) Representative flow cytometry plots (left) and statistics (right) showing the percentage of WT or CD155-KO U937 cell death cocultured at indicated ratios with mock T or CD155 CAR-T cells for 4 hours (*n* = 4 individual donors). (**C**) Mock T or CD155 CAR-T cells were cultured at indicated ratios with MOLM13, U937, or THP-1 cells for 4 hours. Luciferase activity in the wells containing tumor cells was measured with a luminescence microplate reader (*n* = 4 individual donors). (**D**) Representative flow cytometry plots (left) and statistics (right) showing the percentage of IFN-γ^+^ and TNF^+^ T cells in mock T or CD155 CAR-T cells cocultured with WT or CD155-KO MOLM13 cells for 4 hours (*n* = 4 individual donors). (**E**) Representative flow cytometry plots (left) and statistics (right) showing the percentage of IFN-γ^+^ and TNF^+^ T cells in mock T or CD155 CAR-T cells cocultured with WT or CD155-KO U937 cells for 4 hours (*n* = 4 individual donors). IFN-γ was detected using a phycoerythrin-conjugated antibody (IFN-γ–PE), and TNF was detected using a Brilliant Violet 650–conjugated antibody (TNF-BV650) (**D** and **E**). Data represent the mean ± SD and were analyzed by 1-way ANOVA with repeated measures (**D** and **E**) or 2-way ANOVA with repeated measures (**A**–**C**).

To assess the cytolytic activity of CD155 CAR-T cells against AML, we performed flow cytometry–based killing assays. AML cell lines lacking CD155 were used as controls to evaluate the specificity and potential off-target effects of the CD155 CAR (Supplemental Figure 2A). The results demonstrated that, at various effector to targets (E/T) ratios, CD155 CAR-T cells effectively eliminated CD155^+^ MOLM13, U937, and THP-1 cell lines, compared with mock T cells, whereas CD155 CAR-T cells showed no cytotoxicity toward the same AML cell lines having undergone genetic disruption of CD155 (CD155-KO). (Figure 2, A and B, and Supplemental Figure 3A). We further confirmed these findings using luminescence-based killing assays, which produced results consistent with the flow cytometry analysis (Figure 2C). Additionally, we evaluated the ability of CD155 CAR-T cells to produce key cytokines involved in tumor eradication, such as TNF and IFN-γ. CD155 CAR-T cells secreted significantly higher levels of TNF and IFN-γ compared with mock T cells when exposed to CD155^+^ AML cell lines, but not to CD155-KO AML cell lines (Figure 2, D and E, and Supplemental Figure 3B). Collectively, these findings demonstrate that CD155 CAR-T cells specifically target and effectively eliminate CD155-expressing AML cell lines in vitro.

### CD155 CAR-T cells exhibit potent antitumor efficacy against AML cell lines in vivo.

**Figure 3:**
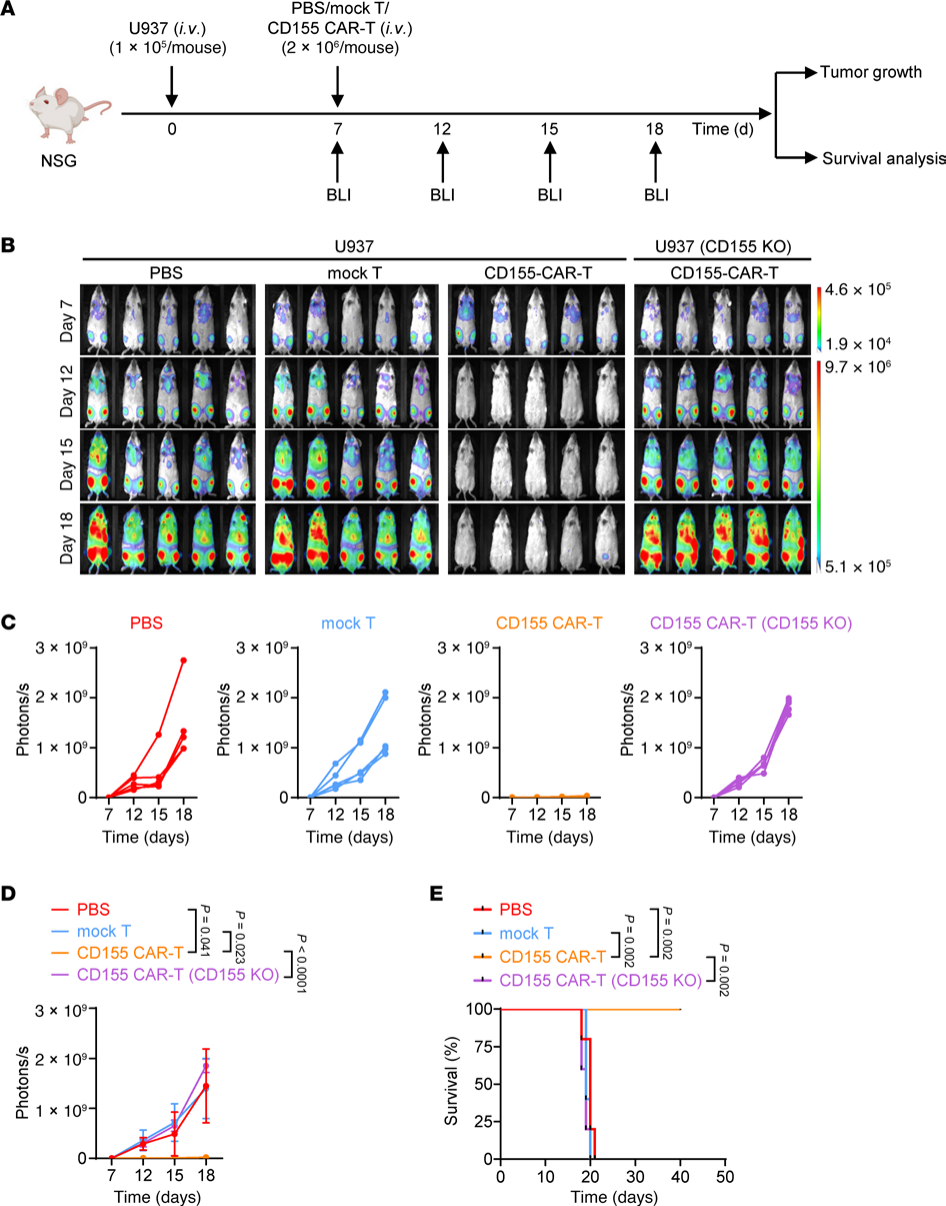
CD155 CAR-T cells exhibit potent antitumor efficacy against U937 in vivo. (**A**) Diagram of the treatment scheme used for in vivo experiments. Wild-type or CD155-KO U937 cells (*n* = 1 × 10^5^) were i.v. injected into NSG mice, followed by an i.v. infusion of indicated number of mock T cells, CD155 CAR-T cells, or PBS (*n* = 5 mice/group) on day 7. (**B**–**D**) Bioluminescence images (**B**), quantification of tumor burden (**C** and **D**), and survival curves (**E**) in U937 tumor-bearing mice after different treatments. Data represent the mean ± SD and were analyzed by 2-way ANOVA with repeated measures (**D**). For Kaplan-Meier survival curves, statistical significance was calculated with a log-rank test (**E**).

We next examined whether CD155 CAR-T cells could inhibit AML growth in vivo. Using an AML model (48), we i.v. injected NOD-scid IL-2Rγ^null^ (NSG) mice with firefly luciferase–labeled (FFLuc-labeled) CD155^+^ or CD155-KO AML cells, followed by an infusion of either PBS, mock T cells, or CD155 CAR-T cells (Figure 3A). Tumor growth was tracked through serial bioluminescence imaging. Our results showed that although PBS and mock T cells failed to suppress the growth of wild-type U937 tumors, CD155 CAR-T cells significantly reduced tumor burden and extended mouse survival compared with both control groups (Figure 3, B–E). Notably, CD155 CAR-T cells had no effect on CD155-KO U937 tumors, confirming that their therapeutic efficacy depends on CD155 expression (Figure 3, B–E). We further validated these findings using FFLuc-labeled CD155^+^ and CD155-KO MOLM13 AML cells in similar experiments (Supplemental Figure 3C). Consistent with the U937 AML model, CD155 CAR-T cells effectively inhibited leukemic growth and improved survival in the CD155^+^ MOLM13 AML model, whereas no effect was observed in the CD155-KO AML model (Supplemental Figure 3, D–F). These findings collectively demonstrate that CD155 CAR-T cells exhibit potent antitumor activity against AML cell lines in vivo.

### CD155 CAR-T cells efficiently lyse primary AML blasts in vitro and in vivo.

**Figure 4:**
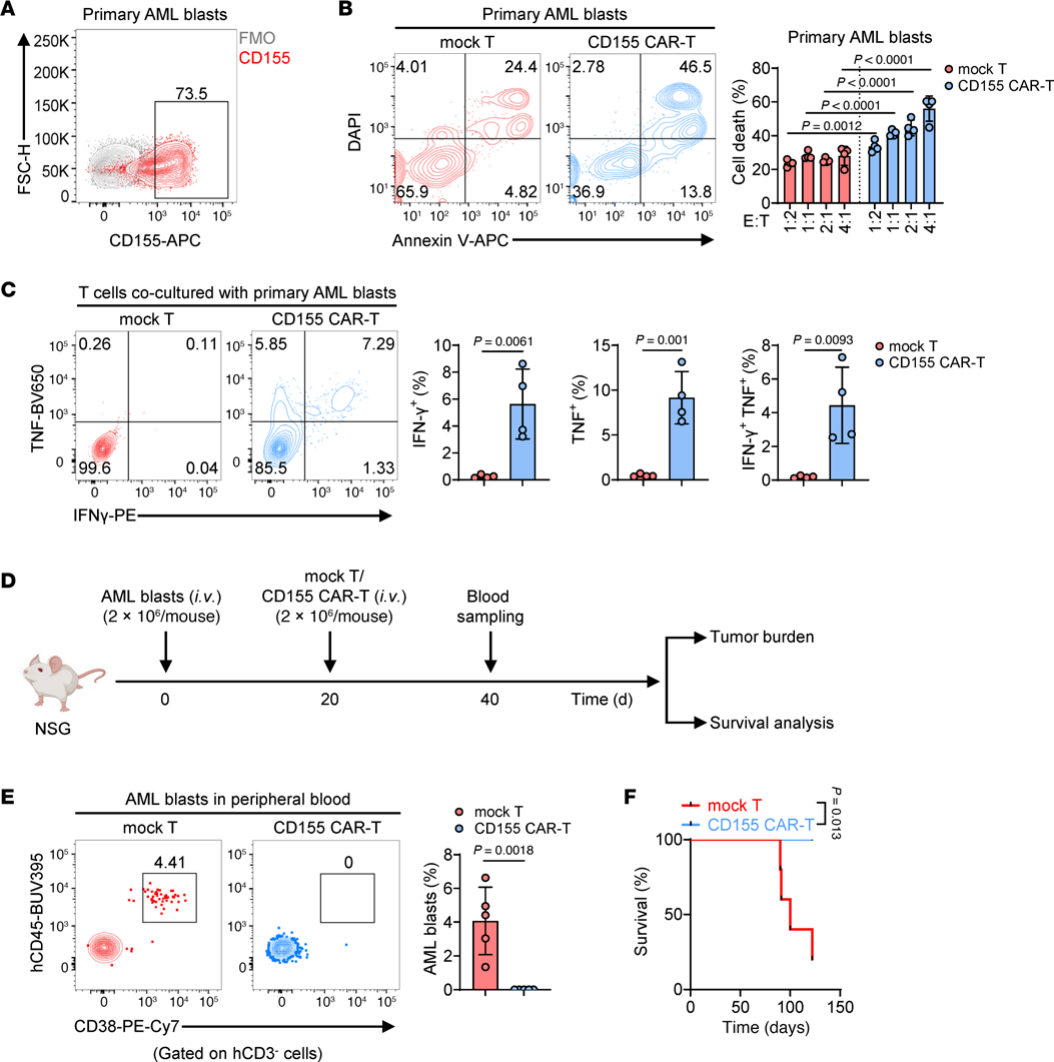
CD155 CAR-T cells efficiently lyse primary AML blasts in vitro and in vivo. (**A**) Representative flow cytometry plots of the percentage showing CD155 expression on primary AML blasts. FSC-H (forward scatter height) was used to assess cell size. (**B**) Representative flow cytometry plots (left) and statistics (right) showing the percentage of primary AML blasts cell death cocultured at indicated ratios with mock T or CD155 CAR-T cells for 4 hours (*n* = 4 individual donors). (**C**) Representative flow cytometry plots (left) and statistics (right) showing the percentage of IFN-γ^+^ and TNF^+^ T cells in mock T or CD155 CAR-T cells cocultured with primary AML blasts for 4 hours (*n* = 4 individual donors). IFN-γ was detected using a phycoerythrin-conjugated antibody (IFN-γ–PE), and TNF was detected using a Brilliant Violet 650–conjugated antibody (TNF-BV650). (**D**) Diagram of the treatment scheme used for in vivo experiments. AML blasts (*n* = 2 × 10^6^) were i.v. injected into NSG mice, followed by an i.v. infusion of indicated number of mock T cells or CD155 CAR-T cells (*n* = 5 mice per group). (**E**) Quantitative analysis of tumor cells in peripheral blood of mice treated with mock T or CD155 CAR-T cells. (**F**) Survival curves of AML-bearing mice after different treatments. Data represent the mean ± SD and were analyzed by 2-way ANOVA with repeated measures (**B**) or 2-tailed Student’s *t* tests (**C** and **E**). For Kaplan-Meier survival curves, statistical significance was calculated with a log-rank test (**F**).

After demonstrating the strong antitumor effects of CD155 CAR-T cells against AML cell lines, we next assessed their cytotoxicity against patient-derived AML blasts. Flow cytometry analysis of blasts from a patient with AML revealed that 73.5% expressed CD155, indicating a substantial target for CD155 CAR-T therapy (Figure 4A). We then exposed these AML blasts to either mock T cells or CD155 CAR-T cells at various E/T ratios. Results from cytometry-based killing assays showed that CD155 CAR-T cells, but not mock T cells, effectively lysed the primary AML blasts in a dose-dependent manner (Figure 4B). Furthermore, CD155 CAR-T cells secreted significantly higher levels of TNF and IFN-γ in response to the AML blasts compared with mock T cells (Figure 4C). To assess the in vivo antitumor efficacy, we used a patient-derived xenograft mouse model. AML blasts from the same patient whose blasts were subjected to flow cytometry analysis were i.v. injected into NSG mice, followed by infusions of either mock T cells or CD155 CAR-T cells (Figure 4D). Analysis of blood from treated mice showed that CD155 CAR-T cells significantly reduced the number of circulating AML blasts compared with mock T cells (Figure 4E). Additionally, mice treated with a single dose of 2 × 10^6^ CD155 CAR-T cells/mouse exhibited a significant extension of survival compared with those receiving mock T cells (Figure 4F). Taken together, these results demonstrate that CD155 CAR-T cells have effective antileukemic activity against primary AML blasts both in vitro and in vivo.

### CD155 CAR-T cells have strong antitumor effects against solid tumors.

**Figure 5:**
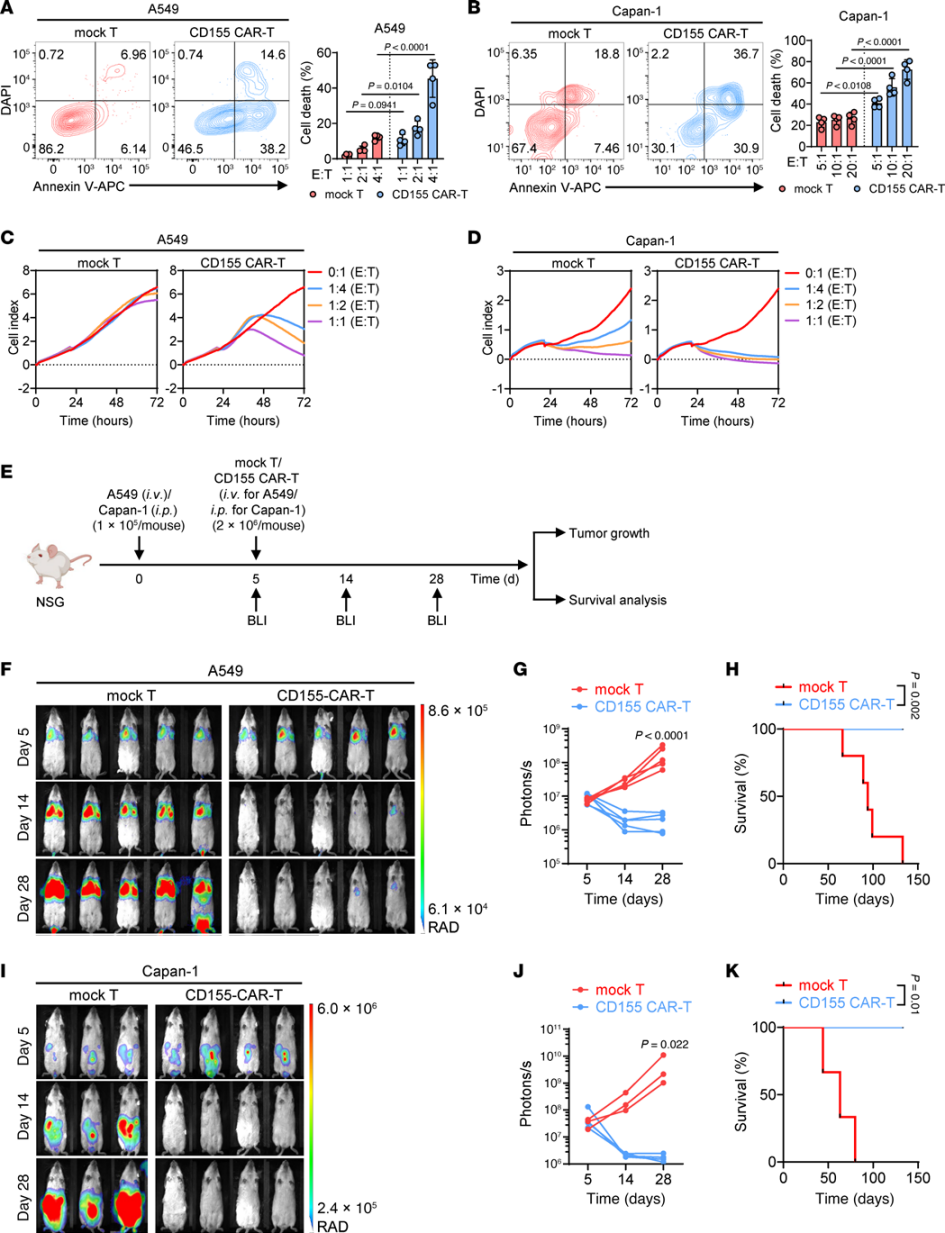
CD155 CAR-T cells show strong antitumor effects against solid tumors. (**A** and **B**) Representative flow cytometry plots (left) and statistics (right) showing the percentage of A549 (**A**) or Capan-1 (**B**) cell death cocultured at indicated ratios with mock T or CD155 CAR-T cells for 4 hours (*n* = 4 individual donors). (**C** and **D**) A549 or Capan-1 cells were cultured at the indicated ratios with mock T or CD155 CAR-T cells for 72 hours. Real-time cell analysis of the cytotoxicity of mock T or CD155 CAR-T cells against A549 (**C**) or Capan-1 (**D**) tumor cells is presented as the growth index of the residual cancer cells (*n* = 4 individual donors). (**E**) Diagram of the treatment scheme used for in vivo experiments. A549 tumor cells (*n* = 1 × 10^5^) were i.v. injected into NSG mice, followed by an i.v. infusion of indicated number of mock T cells or CD155 CAR-T cells. Capan-1 tumor cells (*n* = 1 × 10^5^) were i.p. injected into NSG mice, followed by an i.p. infusion of indicated number of mock T cells or CD155 CAR-T cells. (**F**–**H**) Bioluminescence images (BLIs) (**F**), quantification of tumor burden (**G**), and survival curves (**H**) in A549 tumor-bearing mice after different treatments (*n* = 5 mice per group). (**I**–**K**) Bioluminescence images (**I**), quantification of tumor burden (**J**), and survival curves (**K**) in Capan-1 tumor-bearing mice after different treatments (*n* = 3 mice in mock T group; *n* = 4 mice in CD155 CAR-T group). Data represent the mean ± SD (**A** and **B**) and were analyzed by 2-way ANOVA with repeated measures (**A**, **B**, **G**, and **J**). For Kaplan-Meier survival curves, statistical significance was calculated with a log-rank test (**H** and **K**).

Next, we investigated the efficacy of CD155 CAR-T cells against various solid tumors. CD155 surface expression was measured on several solid tumor cell lines, including A549 (NSCLC), Capan-1 (pancreatic cancer), HepG2 ( liver cancer), U251 (brain cancer), MDA-MB-231 (breast cancer), and HT29 (colon cancer). All the cell lines showed high levels of CD155 expression (Supplemental Figure 4A). To further explore CD155 expression in clinical contexts, we performed IHC staining on human normal and cancerous tissues, including breast, colon, lung, uterus, pancreas, and testis. The results showed that CD155 expression was either absent or modest in normal tissues but was significantly upregulated in their cancerous counterparts, reinforcing the potential of CD155 as a target in solid tumors (Supplemental Figure 4B). To determine the cytotoxic potential of CD155 CAR-T cells against CD155^+^ solid tumors, we cocultured the aforementioned tumor cell lines with either mock T cells or CD155 CAR-T cells at different E/T ratios and conducted cytometry-based killing assays. CD155 CAR-T cells, unlike mock T cells, effectively eradicated the tumor cells in a dose-dependent manner (Figure 5, A and B, and Supplemental Figure 4, C–F). To further confirm these findings, real-time cell analysis assays were performed. We observed that in contrast to mock T cells, CD155 CAR-T cells potently inhibited A549 tumor cell growth in a dose-dependent manner (Figure 5C). Interestingly, although mock T cells exhibited antitumor activity against Capan-1 in a dose-dependent manner, CD155 CAR-T cells were far more potent, showing substantial tumor inhibition even at lower E/T ratios, where mock T cells had minimal effect (Figure 5D). To assess the in vivo antitumor efficacy, NSG mice were injected with FFLuc-labeled A549 (i.v.) or Capan-1 cells (i.p.), followed by treatment with either mock T cells or CD155 CAR-T cells (Figure 5E). Consistent with the in vitro data, administration of CD155 CAR-T cells resulted in significantly reduced tumor burden and extended survival in mice bearing A549 (Figure 5, F–H) and Capan-1 (Figure 5, I–K) tumors, compared with those treated with mock T cells. These results demonstrate that CD155 CAR-T cells exhibit robust antitumor activity across multiple solid tumors both in vitro and in vivo.

### Comparative analysis of specificity, functionality, and DNAM-1/TIGIT binding interference between CD155-CAR-T and CD19-CAR-T cells.

To further validate the specificity and durability of CD155 CAR-T cells, we conducted several additional experiments. First, to clarify the function of our control groups, we note that the mock T cells used in this study were transduced with an empty vector and served as negative controls lacking CAR expression or antigen specificity. In parallel, we generated CD19 CAR-T cells using the same retroviral backbone and signaling domains as CD155 CAR-T cells, serving as an antigen-specific functional control. In vitro cytotoxicity assays revealed that CD155 CAR-T cells, but not mock T cells or CD19 CAR-T cells, effectively killed CD155^+^CD19^–^ tumor cells (U937 and A549) in a dose-dependent manner (Supplemental Figure 5, A–C). CD19 CAR-T cells displayed no activity against these tumors, confirming the antigen-specific cytotoxicity of CD155 CAR-T cells (Supplemental Figure 5, B and C). In vivo, CD155 CAR-T cells also significantly reduced U937 tumor burden compared with CD19 CAR-T cells, further supporting their specificity (Supplemental Figure 5F). To assess the persistence and long-term functionality of CD155 CAR-T cells, we performed a rechallenge assay in which CAR-T cells were exposed to fresh U937 or A549 tumor cells in 3 consecutive rounds over a total of 72 hours. CD155 CAR-T cells retained potent cytolytic activity throughout the assay and consistently outperformed CD19 CAR-T cells under identical conditions (Supplemental Figure 5, D and E). These results highlight the sustained cytotoxic potential of CD155 CAR-T cells and support their promise for long-lasting remission. Consistent with this, CD155 CAR-T treatment led to durable tumor clearance in A549- and Capan-1–bearing mice (Figure 5, F–K). In contrast, although CD155 CAR-T cells significantly reduced tumor burden in the U937 mouse model, some mice exhibited tumor relapse around day 24 after injection (Supplemental Figure 5F). Analysis of residual tumor cells revealed persistent CD155 expression (Supplemental Figure 5G), suggesting that relapse was not due to antigen loss but rather to limited CAR-T cell persistence and/or insufficient dosing. These findings underscore the potential benefit of enhanced dosing strategies, such as multiple infusions, higher cell doses, or combination therapies, in achieving durable remission in aggressive disease settings.

To further compare CD155 and CD19 as tumor antigen targets, we examined the killing activity of both CAR-T cell types against NALM6 cells, a human B-cell leukemia line that co-expresses CD19 and CD155 (44), which was confirmed by using flow cytometry (Supplemental Figure 5H). In vitro cytotoxicity assays demonstrated that both CD155 CAR-T cells and CD19 CAR-T cells efficiently eliminated NALM6 cells in a dose-dependent manner (Supplemental Figure 5I). These findings demonstrate that CD155 CAR-T cells exhibit antitumor efficacy comparable to CD19 CAR-T cells in CD155^+^CD19^+^ leukemia models. Notably, however, only CD155 CAR-T cells are capable of targeting CD155^+^CD19^–^ tumor cells, whereas CD19 CAR-T cells are ineffective in this context (Supplemental Figure 5, B and C). Because most myeloid and solid tumors lack CD19 expression, and CD19^+^ tumors may downregulate CD19 as an escape mechanism (49), these results highlight CD155 as a more broadly applicable target for CAR-T therapy.

Given the critical role of the CD155/TIGIT/DNAM-1 axis in modulating immune responses within the tumor microenvironment, and the potential concern that CAR-T cell engagement of CD155 might interfere with these regulatory pathways, we next investigated whether CD155 CAR-T cells disrupt CD155’s interaction with its native cognate immune ligands. To address this, we performed flow cytometry–based competition assays assessing TIGIT-Fc and DNAM-1-Fc binding to CD155. NALM6 cells were incubated with TIGIT-Fc or DNAM-1-Fc in the presence of either CD155 CAR-T or CD19 CAR-T cells. Binding was quantified by flow cytometry. Our results showed that CD155 CAR-T cells did not interfere with TIGIT or DNAM-1 binding as compared with CD19 CAR-T cells (Supplemental Figure 5, J and K), indicating that the single-chain variable fragment (scFv) used in CD155 CAR-T cells binds to an epitope distinct from those recognized by TIGIT or DNAM-1. These findings suggest that our CD155 CAR-T cells are unlikely to impair endogenous immune regulatory interactions mediated through the CD155/TIGIT or CD155/DNAM-1 axis.

### Generation and functional analysis of humanized CD155 CAR-T cells.

**Figure 6:**
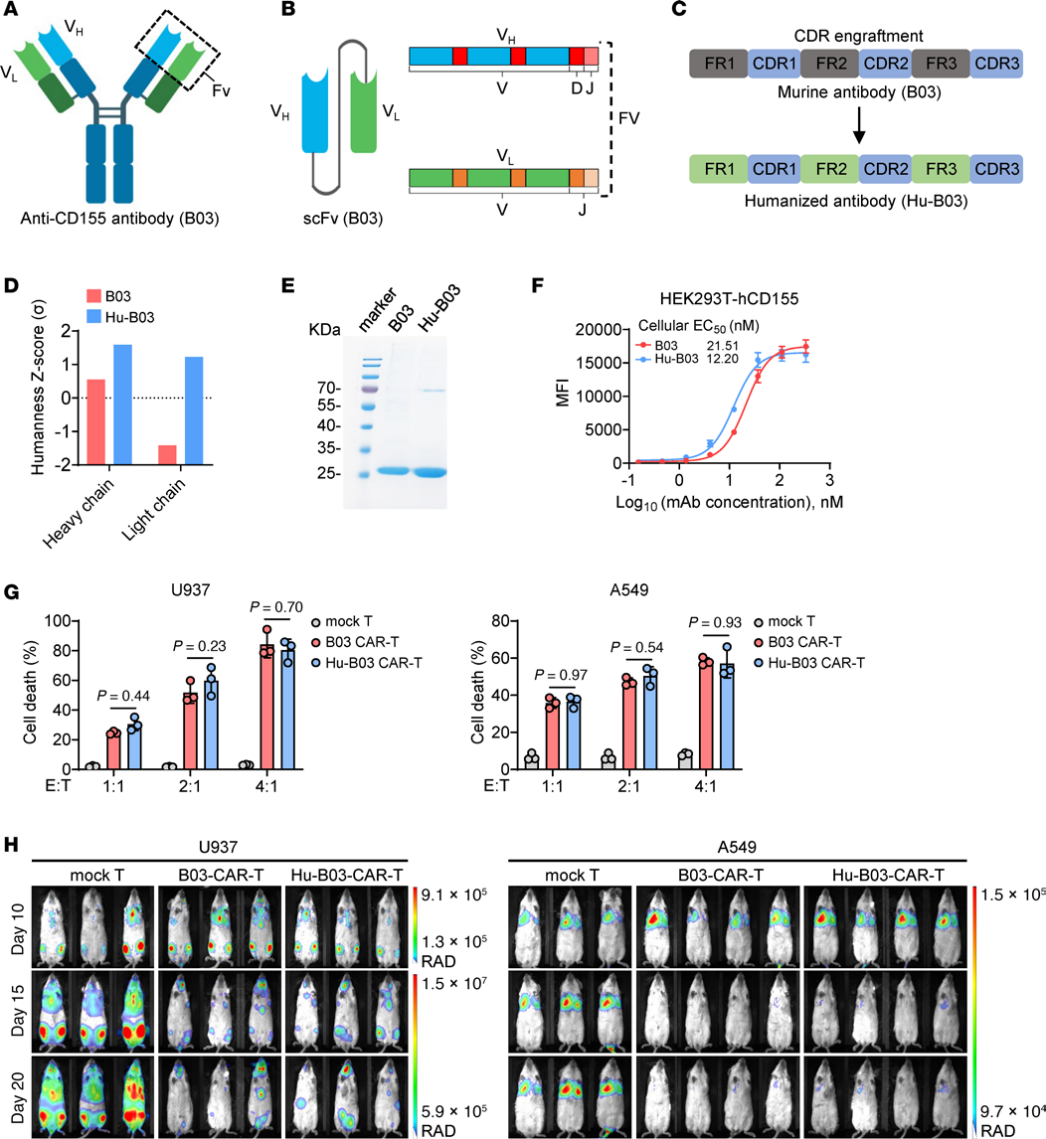
Generation and functional analysis of humanized CD155 CAR-T cells. (**A**) Schematic representation of the murine anti–human CD155 antibody (B03) used in our CAR-T studies. (**B**) Diagram of the B03 scFv derived from the antibody in (**A**). (**C**) Diagram of the CDR engraftment process used to humanize the murine antibody B03 to Hu-B03. (**D**) Results of the humanness *z* score (σ) for the light and heavy chains of both B03 and Hu-B03 antibodies (see Methods). (**E**) Reduced PAGE analysis showing the migration of B03 and Hu-B03 scFvs at approximately 25 kDa. (**F**) Flow cytometry analysis assessing the reactivity of B03 and Hu-B03 scFvs against the HEK293T-hCD155 cell line (*n* = 3 replicates for each scFv). (**G**) Statistics showing the percentage of U937 (left) or A549 (right) cell death cocultured at indicated ratios with mock T, B03 CAR-T, or Hu-B03 CAR-T cells for 4 hours (*n* = 3 individual donors). (**H**) Bioluminescence images in U937 tumor-bearing mice (*n* = 3 mice per group) or A549 tumor-bearing mice (*n* = 3 mice in mock T group; *n* = 4 mice in B03/Hu-B03 CAR-T groups) after different treatments. Data represent the mean ± SD (**F** and **G**) and were analyzed by 2-way ANOVA with repeated measures (**G**). EC_50_ values were determined by fitting a nonlinear 4-parameter dose-response curve (**F**).

In the experiments described thus far, we generated a murine mAb (B03) to create the CAR directed against human (h)CD155 (see Methods). The structure of the parental B03 antibody and its derived scFv used in CAR design are illustrated in Figure 6, A and B. Humanizing CARs derived from murine antibodies is crucial to reduce immunogenicity and improve the safety and efficacy of CAR-T cell and CAR-NK cell therapies in clinical applications (50, 51). Using complementarity-determining region (CDR) grafting and back-mutation (50), we humanized the chimeric CD155 antibody Hu-B03 by incorporating murine CDRs from the original B03 antibody into a human antibody framework (Figure 6C). Humanness analysis (52) revealed that both the heavy and light chains of Hu-B03 had higher humanness *z* scores compared with the original B03, indicating a successful humanization process that enhanced similarity to human antibodies, potentially reducing immunogenicity and improving clinical applicability (Figure 6D).

To validate the binding activity of Hu-B03 against hCD155 and compare it with the original B03, we expressed and purified both Hu-B03 and B03 scFvs in HEK293F cells (Figure 6E). Flow cytometry analysis showed that Hu-B03 scFv bound effectively to HEK293T cells overexpressing hCD155, with an EC_50_ of 12.20 nM, indicating a higher binding affinity than the original B03 scFv, which had an EC_50_ of 21.51 nM (Figure 6F). Based on the design of the B03 scFv CAR, we constructed a second-generation CAR incorporating the Hu-B03 scFv, along with the 4-1BB costimulatory domain and the CD3ζ signaling domain (Supplemental Figure 6A). Hu-B03 CAR-T cells were generated by retroviral transduction, achieving CAR-positive rates comparable to those of B03 CAR-T cells (Supplemental Figure 6B). Next, we assessed the in vitro cytotoxicity of Hu-B03 CAR-T cells against AML and solid tumor cells using flow cytometry–based killing assays. Hu-B03 CAR-T cells exhibited dose-dependent killing activity, comparable to that of B03 CAR-T cells in vitro (Figure 6G and Supplemental Figure 6, C–E). To further evaluate their antitumor efficacy in vivo, we used tumor models in which FFLuc-labeled U937 or A549 cells were i.v. injected into NSG mice, followed by infusions of mock T cells, B03 CAR-T cells, or Hu-B03 CAR-T cells (Supplemental Figure 6F). Administration of Hu-B03 CAR-T cells resulted in significantly reduced tumor burden in both U937 AML and A549 NSCLC mouse models, demonstrating efficacy comparable to B03 CAR-T cells (Figure 6H). These findings collectively suggest that Hu-B03 CAR-T cells maintained strong antitumor efficacy against CD155^+^ AML and NSCLC in vitro and in vivo while potentially reducing immunogenicity, thereby enhancing their suitability for clinical applications.

### Comprehensive evaluation of CD155 expression suggests a tolerable off-tumor safety profile for CD155 CAR-T therapy.

To ensure the clinical applicability and safety of CD155 CAR-T therapy, we conducted a thorough evaluation of CD155 expression in various hematopoietic and nonhematopoietic normal human tissues to assess potential on-target, off-tumor effects. Our analysis revealed minimal CD155 expression on lymphoid-derived immune cells, including CD4^+^ T cells, CD8^+^ T cells, B cells, and NK cells isolated from PBMCs (Supplemental Figure 7, A and B). In contrast, myeloid-derived cells, such as monocytes and conventional DCs, exhibited high levels of CD155, with neutrophils showing moderate expression (Supplemental Figure 7, A and B). Given the expression of CD155 in mature myeloid cells, we undertook further evaluation of its expression across early HSPCs populations using flow cytometry on CD34^+^ HSPCs isolated from healthy human donors. CD155 expression was minimal or undetectable in primitive subsets, including hematopoietic stem cells (HSCs), multipotent progenitors, and multilymphoid progenitors, as indicated by low frequencies of CD155^+^ cells and low MFI (Supplemental Figure 7C). Modest increases in CD155 expression were observed in common myeloid progenitors (CMPs), whereas higher proportions of CD155^+^ cells were detected in granulocyte-macrophage progenitors (GMPs) and megakaryocyte-erythroid progenitors (MEPs) (Supplemental Figure 7C). Notably, even among these more differentiated populations, CD155 expression levels were variable and generally low, with the exception of 1 donor (donor 2), whose GMPs exhibited 60.9% CD155 positivity. In other donors, CD155^+^ frequencies ranged from 1.1% to 38.2% (Supplemental Figure 7C). These findings suggest CD155 CAR-T cells are unlikely to target primitive HSCs but may engage a subset of more differentiated myeloid progenitors. The observed donor-to-donor variability, particularly within CMP and GMP populations, underscores the importance of considering individual patient profiles when evaluating the risk of on-target, off-tumor hematopoietic toxicity in future clinical applications.

To further assess potential on-target, off-tumor effects, we analyzed CD155 expression patterns alongside reference genes (*EGFR*, *ERBB2*, and *ROR1*), which are currently being investigated in clinical trials for CAR-T therapies (9). Using scRNA-Seq data from healthy human tissues, we depicted a comprehensive Cross-Organ Off-Target Transcriptomic Atlas (53). This data set includes tissues from the brain, colon, heart, kidney, liver, lung, muscle, spleen, and testis (Supplemental Table 1). Our analysis showed that EGFR had high expression across key tissues, including the brain, heart, kidney, liver, lung, and muscle (Supplemental Figure 8A). ERBB2 was prominently expressed in the kidney and lung, whereas ROR1 exhibited high levels in the heart, kidney, and alveolar type 1 cells in the lung (Supplemental Figure 8A). In contrast, CD155 displayed relatively low and restricted expression across healthy tissues, mainly localized to endothelial and epithelial cells (Supplemental Figure 8A). This limited expression pattern suggests that CD155 may have a lower risk of on-target, off-tumor effects when compared with EGFR, ERBB2, and ROR1. To complement these transcriptomic findings, we also performed IHC staining of CD155 in normal human tissues to validate protein-level expression across organs. Consistent with the scRNA-Seq data, IHC analysis revealed low to modest CD155 expression in the spleen, lung, kidney, brain, and colon (Supplemental Figure 8B). Furthermore, IHC staining of tissues that were not included in the scRNA-Seq analysis, such as the stomach, ovary, pancreas, prostate, skin, and uterus, also showed low to modest CD155 expression (Supplemental Figure 8B). Notably, relatively higher CD155 expression was observed in liver tissue, which may warrant further investigation (Supplemental Figure 8B). These results provide additional evidence that CD155 expression is largely limited in normal tissues and support its candidacy as a therapeutic target with a potentially reduced risk of widespread off-tumor toxicity.

### Hematopoietic safety assessment of humanized CD155 CAR-T cells.

**Figure 7:**
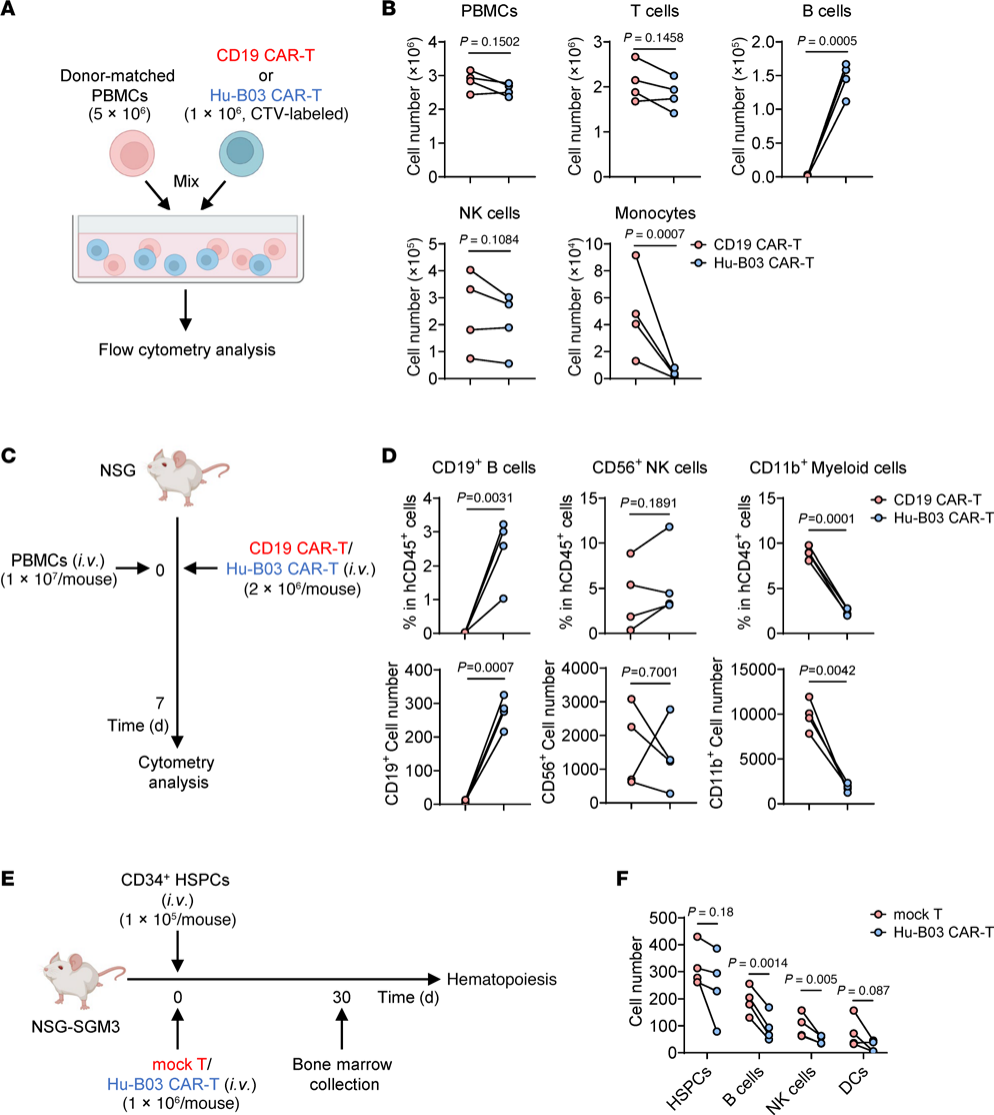
Hematopoietic safety assessment of humanized CD155 CAR-T cells. (**A**) Schematic of the in vitro experimental design. A total of 5 × 10^6^ donor-matched PBMCs were cocultured with 1 × 10^6^ CellTrace Violet (CTV)-labeled CD19 CAR-T cells or Hu-B03 CAR-T cells for 12 hours (*n* = 4 individual donors per group). Flow cytometry–based cytotoxicity assays were performed to assess cell death across immune cell subsets. (**B**) Quantification of total PBMCs and specific immune subsets (T cells, B cells, NK cells, and monocytes) after coculture with CD19 CAR-T or Hu-B03 CAR-T cells. (**C**) Schematic of the in vivo experimental design. A total of 1 × 10^7^ PBMCs were injected into NSG mice concurrently with 2 × 10^6^ CD19 CAR-T cells or Hu-B03 CAR-T cells (*n* = 4 individual donors per group). Mice were sacrificed on day 7 after injection for bone marrow analysis. (**D**) Quantification of the frequency and absolute number of B cells, NK cells, and myeloid cells in bone marrow from treated mice. (**E**) Schematic of the in vivo experimental design. A total of 1 × 10^5^ CD34^+^ HSPCs were transplanted into NSG-SGM3 mice concurrently with the indicated number of either mock T cells or Hu-B03 CAR-T cells (*n* = 4 individual donors per group). Mice were sacrificed on day 30 after injection for analysis of human CD34^+^ HSPCs and their differentiation, including mature lymphoid and myeloid populations in the bone marrow. (**F**) Quantification of human CD34^+^ HSPCs, CD19^+^ B cells, CD56^+^ NK cells, and CD11c^+^ DCs in bone marrow from mice treated with mock T cells or Hu-B03 CAR-T cells. Data represent the mean ± SD and were analyzed by paired 2-tailed Student’s *t* tests (**B**, **D**, and **F**).

To further support the safety profile of humanized CD155 CAR-T (Hu-B03 CAR-T) cells and address concerns regarding potential hematologic toxicity, we conducted additional functional assessments using both in vitro and in vivo models. First, we compared cytotoxic selectivity in vitro by coculturing donor-matched human PBMCs with either CD19 CAR-T or Hu-B03 CAR-T cells. Survival of immune subsets was assessed by flow cytometry (Figure 7A). CD19 CAR-T cells selectively depleted B cells while sparing other populations (Figure 7B). In contrast, Hu-B03 CAR-T cells specifically reduced monocytes, with minimal effects on T cells, B cells, or NK cells (Figure 7B). These distinct, target-restricted profiles are consistent with known antigen expression patterns of CD19 (3) and CD155 (Supplemental Figure 7A). We next validated these findings in vivo using a PBMC-engrafted NSG mouse model (Figure 7C). Flow cytometric analysis of bone marrow on day 7 after injection showed that CD19 CAR-T cells depleted B cells, whereas Hu-B03 CAR-T cells primarily reduced CD11b^+^ myeloid cells (Figure 7D), confirming their in vivo specificity.

To assess potential toxicity to HSPCs, we performed additional in vivo studies using a human HSPC-engrafted NSG-SGM3 model, which expresses human IL-3, GM-CSF, and SCF to support human hematopoiesis. CD34^+^ HSPCs from healthy donors were transplanted into NSG-SGM3 mice concurrently with either mock T cells or Hu-B03 CAR-T cells (Figure 7E). Flow cytometric analysis of bone marrow on day 30 revealed significantly reduced NK and B cells in Hu-B03 CAR-T–treated mice compared with controls, along with nonsignificant decreases in CD34^+^ HSPCs and DCs (Figure 7F). These reductions across multiple hematopoietic subsets likely reflect low-level antigen recognition by Hu-B03 CAR-T cells. This is consistent with our prior observations showing partial CD155 expression on HSPCs. Importantly, a population of CD34^+^ HSPCs remained detectable and retained multilineage differentiation capacity, suggesting that hematopoietic function was at least partially preserved (Figure 7F). Taken together, these findings indicate that although Hu-B03 CAR-T therapy may affect certain aspects of hematopoiesis, it does not eliminate HSC function, potentially reducing the need for hematopoietic stem cell transplantation (HSCT) after Hu-B03 CAR-T cell treatment. This supports its clinical potential with appropriate monitoring and risk management strategies.

### Characterization of mCD155 CAR-T cells.

Because Hu-B03 CAR-T cells do not recognize murine CD155 (data not shown), they are not suitable for evaluating systemic on-target, off-tumor effects in mouse models. Indeed, sequence alignment analysis revealed limited conservation between human and mouse CD155 proteins, with only 43.97% sequence identity and 56.68% similarity (Supplemental Figure 9A), indicating antibodies recognizing human CD155 are unlikely to cross-react with mouse CD155. To address this limitation and comprehensively evaluate the safety of targeting CD155, we generated anti–mouse CD155 CAR-T cells and assessed their function and safety in fully immunocompetent syngeneic mouse models. After developing a CAR specific to murine CD155 (see Methods), we examined the functional efficacy of mCD155 CAR-T cells, focusing on their ability to kill mCD155-expressing tumor cells. We first confirmed high mCD155 expression on the murine pancreatic tumor cell line KPC (Supplemental Figure 10A). KPC cells were then cocultured with either mock T cells or mCD155 CAR-T cells at various E/T ratios, and cytometry-based killing assays were performed. The results showed that mCD155 CAR-T cells, but not mock T cells, efficiently lysed KPC tumor cells in a dose-dependent manner (Supplemental Figure 10B).

To further evaluate the specificity and off-target effects of mCD155 CAR-T cells, we used the C1498 murine AML cell line to create mCD155-KO C1498 AML cells using CRISPR-Cas9 (Supplemental Figure 10C). CD155^+^ and CD155-KO C1498 cells were then cocultured with either mock T cells or mCD155 CAR-T cells, followed by cytometry-based killing assays. As expected, mCD155 CAR-T cells efficiently eliminated CD155^+^ C1498 cells, compared with mock T cells, while showing minimal cytotoxicity against CD155-KO C1498 cells (Supplemental Figure 10D). To assess the in vivo antitumor efficacy, C57BL/6 mice were i.p. injected with FFLuc-labeled KPC cells and subsequently treated with either mock T cells or mCD155 CAR-T cells (Supplemental Figure 10E). Consistent with the in vitro findings, mCD155 CAR-T cells significantly reduced tumor burden (Supplemental Figure 10F) and extended survival (Supplemental Figure 10G) in KPC tumor–bearing mice compared with those treated with mock T cells.

To further validate the antigen specificity of CD155 CAR-T cells, we performed analogous experiments using murine CD19 CAR-T cells as functional controls. Consistent with our human CAR-T data, mCD155 CAR-T cells exhibited robust and specific cytotoxicity against CD19^–/low^ CD155^+^ tumor cells such as KPC and C1498, whereas mCD19 CAR-T cells showed minimal activity (Supplemental Figure 10, H and I), further confirming the antigen-specific activity of mCD155 CAR-T cells. Taken together, these results demonstrate that mCD155 CAR-T cells specifically target and eliminate mCD155-expressing tumor cells both in vitro and in vivo, providing robust evidence for their therapeutic potential and suitability for evaluation of on-target, off-tumor effects in immunocompetent mouse models.

### Murine CD155 expression profiles across hematopoietic and normal tissues.

To inform the subsequent safety evaluation of mCD155 CAR-T cells in immunocompetent mouse models, we first assessed the expression patterns of CD155 in murine hematopoietic and normal tissues. Using flow cytometry, we analyzed CD155 expression on both mature and early hematopoietic cell populations in mice. Approximately 20% of mouse T cells expressed CD155, whereas B cells, NK cells, and mature myeloid subsets such as DCs, macrophages, monocytes, and neutrophils showed minimal expression (Supplemental Figure 11A). In contrast to our human data, CD155 expression was more prominent in early hematopoietic compartments, with moderate levels detected on Lineage^–^Sca1^+^c-Kit^+^ (LSK) cells, common myeloid progenitors (CMPs), and MEPs and particularly high levels on granulocyte-monocyte progenitors (GMPs) (Supplemental Figure 11B).

To determine whether CD155 is similarly restricted in nonhematopoietic tissues, we next performed IHC staining of multiple organs using a commercially available anti–mouse CD155 antibody (clone 305; Invitrogen) validated for IHC applications. Unexpectedly, we observed widespread moderate to high CD155 expression across multiple tissues, including the heart, liver, spleen, lung, kidney, and brain (Supplemental Figure 11C). This pattern was markedly different from the expression profile previously observed in human tissues. Interestingly, when we stained the same tissues using our in-house G06 antibody, which is the exact clone used to construct mCD155 CAR-T cells, the staining intensity was minimal or undetectable (Supplemental Figure 11D). These findings raised the possibility that differences in binding affinity between the 2 antibody clones may account for the observed discrepancy. To confirm that the G06 antibody is compatible with IHC and capable of recognizing CD155 in situ, we stained murine tumors that we previously found expressed CD155, such as MC38 and B16F10 (Supplemental Figure 11E), and we observed strong positive signals (Supplemental Figure 11F). This suggests that the G06 antibody may preferentially bind tumor-associated CD155 rather than its form expressed in normal tissues. This property may be beneficial for reducing off-tumor toxicity. Notably, low-affinity CARs have been used in prior studies as a strategy to limit toxicity while retaining tumor-killing activity (54). These findings highlight the importance of using G06-derived CAR-T cells for preclinical safety evaluation in mice, particularly given the broader expression of CD155 in mouse tissues compared with humans.

### mCD155 CAR-T cells show a favorable systemic and neurotoxic safety profile in immunocompetent mouse models.

**Figure 8:**
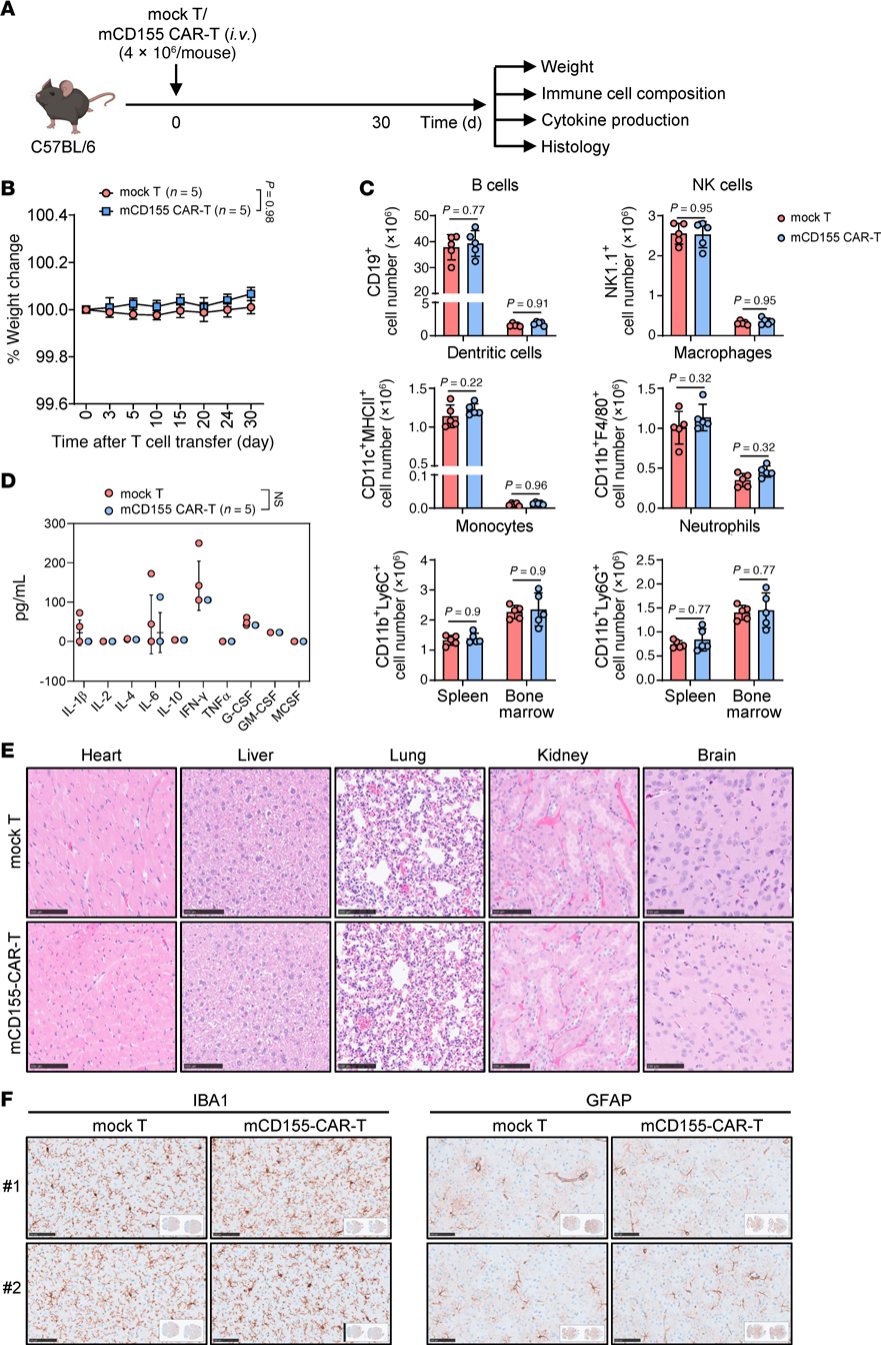
mCD155 CAR-T cells show a favorable systemic and neurotoxic safety profile in immunocompetent mouse models. (**A**) Treatment schedule for in vivo toxicity assessment of mCD155 CAR-T cells in a syngeneic immunocompetent mouse model. (**B**) Weight change of mice injected with mock T or mCD155 CAR-T cells (*n* = 5 mice per group). (**C**) Statistics of absolute cell numbers of CD19^+^ B cells, NK1.1^+^ NK cells, CD11c^+^MHC-II^+^ DCs, CD11b^+^F4/80^+^ macrophages, CD11b^+^Ly6C^+^ monocytes, and CD11b^+^Ly6G^+^ neutrophils in the spleen and bone marrow of mice on day 30 after injection with mock T or mCD155 CAR-T cells (*n* = 5 mice per group). (**D**) Assessment of serum cytokine levels from mice described in (**C**). (**E**) H&E staining of the organs collected from mice described in (**C**). Scale bars: 100 μm. (**F**) IHC analysis of IBA1 (left) and GFAP (right) expression on brain tissues from mice described in (**C**). Scale bars: 100 μm. Data represent the mean ± SD and were analyzed by 2-way ANOVA with repeated measures (**B**) or 2-tailed Student’s *t* tests (**C** and **D**).

Given the broad CD155 expression pattern observed in murine tissues using the commercial antibody, we reasoned that if CD155 CAR-T cells using the G06 clone demonstrate a favorable safety profile under these stringent preclinical conditions, the safety margin would be even more favorable in humans, in whom CD155 expression is more restricted. To test this, we evaluated the safety of G06-based mCD155 CAR-T cells in fully immunocompetent C57BL/6 mice. Mice were treated with a 4-fold higher dose of either mock T cells or mCD155 CAR-T cells compared with that used in the previously described KPC tumor model, followed by a comprehensive safety assessment (Figure 8A). Our results demonstrated that mice injected with mCD155 CAR-T cells exhibited no significant weight loss compared with controls (Figure 8B). Flow cytometry analysis on day 30 revealed no adverse effects on spleen or bone marrow cellularity (Figure 8C), and serum cytokine levels remained within normal ranges (Figure 8D). Histopathological examination of major organs using H&E staining revealed no signs of inflammation or tissue damage (Figure 8E). Additionally, IHC analysis using the G06 anti–mCD155 antibody showed minimal reactivity across all examined organs in both treatment groups (Supplemental Figure 11G). Given that systemic CAR-T cell therapy raises concerns about potential central nervous system toxicity, particularly in clinical settings (55), we next examined brain tissues from the treated mice. IHC staining for IBA1 and GFAP, markers of microglial and astrocyte activation, respectively, revealed no substantial differences between the groups (Figure 8F), indicating no detectable neuroinflammation or damage after mCD155 CAR-T cell therapy. Together, these results support a favorable safety profile for mCD155 CAR-T cells and suggest that even in a setting of relatively broad CD155 expression, low-affinity CAR-T cells derived from the G06 clone do not induce marked on-target, off-tumor toxicity or neurotoxicity.

## Discussion

In this study, we developed and evaluated CD155-targeted CAR-T cells against both human and murine CD155 (hCD155 and mCD155, respectively), demonstrating potent antitumor efficacy in human AML and various human solid tumors. These findings underscore CD155 as a promising target for CAR-T cell therapy, addressing the unmet need in AML and solid tumors where current CAR-T therapies have not yet matched the success observed in B-cell malignancies. Importantly, we identified CD155 as a target of CAR-T cells for treating both AML and solid tumors.

The overexpression of CD155 in AML cell lines, primary AML blasts, as well as its expression in lung, pancreatic, liver, and brain tumors, highlights its broad applicability as a therapeutic target. Notably, CD155 is highly prevalent in LSCs, known to drive AML persistence and relapse (56), making it an attractive target for potentially inducing long-lasting remissions. Previous research has largely focused on the role of CD155 in tumor immune evasion through the CD155/DNAM-1/TIGIT axis, with less attention on its potential as a direct therapeutic target (33). Our work takes this in a different direction by demonstrating that CD155 can be directly targeted with CD155 CAR-T cells in preclinical models, demonstrating feasibility and effectiveness for both AML and solid tumor malignancies.

The dual applicability of CD155 CAR-T cells to both AML and solid tumors could be particularly valuable for patients with secondary malignancies induced as the result of chemotherapy or radiotherapy of a primary tumor, or in patients with certain germline mutations such as *BRCA1* or *BRCA2* that lead to susceptibility to more than 1 type of cancer, sometimes occurring concurrently. Moreover, this approach has the potential to reduce the high costs of CAR-T cell manufacturing. For example, in current autologous CAR-T cell therapy, the same CD155 CAR-expressing virus can be manufactured as a single lot to be used for the treatment of both AML and a variety of solid tumors. For allogeneic cell therapy, such as universal CAR-T cells under investigation and CAR NK cells, which are currently being widely tested in the clinic, this approach can more substantially reduce costs, because “1 cell product fits most.”

A critical step in moving toward clinical translation has been our development of humanized CD155 CAR-T cells (Hu-B03). By reducing the immunogenicity of murine-derived CAR constructs, humanized CARs improve the safety and efficacy of these therapies for human use (50, 51). Our results show that Hu-B03 CAR-T cells retain their potent antitumor activity with comparable efficacy to the original murine B03 CAR-T cells in AML and solid tumor models. Furthermore, we developed a CAR directed against mCD155 and demonstrated in an immunocompetent syngeneic mouse model that the on-target, off-tumor toxicity profile following the administration of mCD155 CAR-T cells appears acceptable. These data, as well as the planned pre–investigative new drug studies in the syngeneic immunocompetent model and using humanized hCD155 CAR-T cells, should allow them to be readily adapted for clinical testing in patients with either advanced AML or advanced solid tumors, or both.

Although our preclinical studies support the overall safety of CD155 CAR-T therapy, careful consideration is still needed regarding potential on-target, off-tumor effects (57). CD155 expression on human myeloid-derived cells, especially monocytes and DCs, raises concerns regarding potential hematologic toxicity. Indeed, our in vitro and in vivo assays demonstrated that CD155 CAR-T treatment significantly reduced myeloid cell populations while sparing lymphocyte subsets, including B cells and NK cells. Additionally, we observed partial CD155 expression on HSPCs. Consistent with this observation, in vivo assays using human CD34^+^ HSPCs demonstrated that Hu-B03 CAR-T cells slightly reduced the number of HSPCs yet preserved sufficient hematopoietic function to potentially avoid the need for HSCT, a frequent requirement for CD33– or CD123–CAR-T therapies in patients with AML (58, 59). However, partial depletion of lymphoid subsets, such as NK and B cells, was observed, necessitating careful long-term monitoring in clinical applications. Should persistent cytopenia develop, incorporating a safety switch, such as truncated EGFR, could provide a clinically relevant mechanism to mitigate toxicity and maintain hematopoietic integrity (60–62).

Furthermore, cross-species differences in CD155 expression must be considered. Although human tissues generally show low to modest CD155 expression, murine tissues stained with a commercial antibody (clone 305) exhibited widespread expression. In contrast, our G06 antibody, which was used to construct mCD155 CAR-T cells, showed minimal reactivity in normal mouse tissues but retained strong binding to CD155^+^ tumor cells. This suggests that the G06 clone may preferentially recognize tumor-associated CD155. These clone-specific binding differences likely account for the absence of systemic toxicity observed in mCD155 CAR-T–treated mice. Additionally, CD155 expression patterns vary across developmental stages and between species, with higher expression in early murine myeloid progenitors compared with mature subsets. Together, these findings highlight the importance of incorporating both murine and humanized models to comprehensively evaluate on-target, off-tumor risks and to improve prediction of human-specific toxicities.

Although CD155 is expressed at low levels in healthy tissues, it is regarded as a stress-induced molecule (63). This function becomes particularly relevant in patients undergoing chemotherapy, because certain chemotherapeutic agents can induce CD155 expression (63). Although this effect could exert a synergistic cytotoxic effect between chemotherapy and CD155 CAR-T cells on tumor cells, it may also increase the risk of on-target, off-tumor toxic effects due to increased CD155 expression on healthy cells. Conversely, other chemotherapeutic agents decrease CD155 expression on tumor cells (64), which could reduce the susceptibility of tumors to CD155 CAR-T cell therapy and contribute to immune evasion. These findings underscore the importance of future studies to characterize the expression patterns and kinetics of CD155 in patients with cancer who are receiving chemotherapy, ensuring that the therapeutic window for CD155 CAR-T cells remains optimal while minimizing risks.

Another important area for investigation involves exploring potential resistance mechanisms that may develop during CD155-targeted therapy, especially considering CD155’s involvement in immune evasion. It is possible that combining CD155 CAR-T cells with other immunomodulatory agents, such as checkpoint inhibitors (65, 66), could enhance response durability and potentially mitigate resistance. Further studies are needed to assess the effectiveness of these combination strategies and to better understand how they might optimize therapeutic outcomes without triggering serious toxicity.

In summary, we show that CD155 CAR-T cells represent a promising therapeutic approach for both AML and solid tumors, including lung cancer and pancreatic cancer. Their potent antitumor efficacy, coupled with early evidence of a favorable safety profile, should allow CD155 CAR-T cells to be considered a valuable addition to the current landscape of cancer cellular immunotherapy. Future efforts should focus on further optimizing the CAR construct and conducting comprehensive safety evaluations to advance this reagent to early-phase clinical trials.

## Methods

### Sex as a biological variable.

Our study examined male and female animals, and similar findings are reported for both sexes.

### Mice.

NSG, NOD.Cg-*Prkdc^scid^ Il2rg^tm1Wjl^* Tg (CMV-IL3, CSF2, KITLG)1Eav/MloySzJ (NSG-SGM3) mice, and wild-type C57BL/6J mice were purchased from Jackson Laboratory; BALB/c mice were obtained from GemPharmatech; and New Zealand rabbits were obtained from Chongqing Sishuo Biotechnology Co. All animals were housed and maintained at the Animal Resource Center at City of Hope or the Laboratory Animal Center of the Chongqing International Institute for Immunology. They were kept under a 12-hour light/dark cycle at temperatures between 18°C and 23°C, with 40%–60% humidity. For mouse studies, both male and female mice, aged 6–8 weeks, were randomly assigned to experimental groups, unless otherwise noted. Experimenters were blinded during survival observations.

### Cell lines.

Human AML cell lines (wild-type and CD155-KO MOLM13, U937, and THP-1), acute lymphocytic leukemia cell line NALM6, and murine AML cell lines (wild-type and CD155-KO C1498) were cultured in RPMI 1640 medium (Gibco) supplemented with 10% FBS (Gibco). These wild-type cell lines were obtained from the American Type Culture Collection (ATCC) or the Leibniz Institute–German Collection of Microorganisms and Cell Cultures. All AML cell lines expressed luciferase. CD155-KO human AML cell lines were generated as previously described (48). The CD155-KO C1498 cell line was created by knocking out the *CD155* gene using CRISPR-Cas9. Specifically, CD155 sgRNA (5′-TCGTCCAGGAGGGTGACCAT-3′) from Integrated DNA Technologies was electroporated into C1498 cells using the Amaxa P3 Primary Cell 4D-Nucleofector X Kit (Lonza, V4XP-3032), following the manufacturer’s protocol. Flow cytometry sorting was subsequently used to isolate CD155-KO cells, establishing a stable cell line. Human solid tumor cell lines, including A549 (NSCLC), Capan-1 (pancreatic cancer), HepG2 (liver cancer), U251 (brain cancer), MDA-MB-231 (breast cancer), and HT29 (colon cancer), along with the murine pancreatic cancer cell line KPC, were cultured in DMEM GlutaMAX medium supplemented with 10% FBS. These cell lines were either purchased from ATCC or obtained from E. Antonio Chiocca’s laboratory at Harvard University (Cambridge, Massachusetts, United States). HEK293T-h/mCD155 cell lines were generated via lentivirus-mediated gene transfer and cultured in DMEM GlutaMAX media with 10% FBS (67, 68). The HEK293T cell line was originally obtained from ATCC, and HEK293F was purchased from Kairui Biotech, where it was cultured in KPM serum-free medium (Kairui Biotech, K03125). All cell lines were routinely tested for *Mycoplasma* contamination using the MycoAlert Plus Mycoplasma Detection Kit (Lonza). All cell culture media were supplemented with 1% penicillin/streptomycin (Gibco), and cultures were maintained at 37°C in a humidified atmosphere with 5% CO_2_.

### Single hCD155/mCD155–specific antibody-secreting cell selection.

To screen for antibody-secreting cell (ASC) selection, the Berkeley Lights Beacon system (69) was used. Female BALB/c mice (aged 6–8 weeks) and male New Zealand rabbits (aged 10–14 weeks) were immunized with the recombinant extracellular domains of human CD155 (hCD155; Sino Biological Inc., 10109-H08H) and mouse CD155 (mCD155; Sino Biological Inc. 50259-M08H), respectively (Supplemental Figure 12, A and B). CD138^+^ plasma cells from hCD155-immunized BALB/c mice and activated ASCs from mCD155-immunized rabbits were automatically loaded onto OptoSelect 11k chips in a plasma blast survival medium that promotes antibody secretion and preserves cell viability (Supplemental Figure 12, C and D) (Berkeley Lights, 75002051). Single-cell penning was performed using opto-electropositioning technology, which uses light to precisely transfer B cells into individual nanoliter chambers, known as NanoPens (Supplemental Figure 12, C and D). This process allowed thousands of ASCs to be positioned into pens across multiple chips within each workflow.

To identify antibodies binding to h/mCD155, an on-chip fluorescence-based assay was conducted. Conjugated beads were prepared by coupling biotinylated h/mCD155 protein (dark gray in Supplemental Figure 12E) to streptavidin-coated assay beads (light gray in Supplemental Figure 12E, left) (Berkeley Lights, 520-00053). These beads were mixed with either a fluorescently labeled anti–mouse secondary antibody (AF568; Thermo Fisher, A-11031) or an anti–rabbit secondary antibody (AF488; Jackson ImmunoResearch, 111-546-144) at a 1:100 dilution and loaded onto OptoSelect 11k chips. Antigen-specific antibodies (black Y-shaped in Supplemental Figure 12E, left) bound to the h/mCD155-conjugated beads, capturing the fluorescent secondary antibodies (red in Supplemental Figure 12E, left). NanoPens adjacent to the fluorescent beads were identified as containing cells secreting antigen-specific antibodies (Supplemental Figure 12E, right). The antigen-specific cells were then exported from the corresponding NanoPen chambers into individual wells of 96-well reverse transcription PCR (RT-PCR) plates containing lysis buffer (Qiagen, 1070498).

### Single B cell sequencing and scFv cloning.

After exportation from the Beacon system, the heavy- and light-chain sequences of antibodies secreted by B cells specific to h/mCD155 were amplified and recovered using the Opto Plasma B Discovery cDNA Synthesis Kit (Berkeley Lights, 750-02030). RNA from each single B cell was purified and isolated using Agencourt RNAClean XP Beads (Beckman Coulter, A63987), followed by a 9 μL RT reaction. First-strand cDNA synthesis and total cDNA amplification were then performed according to the manufacturer’s instructions (Berkeley Lights, 750-02030). The amplified cDNA was purified with Agencourt AMPure XP Beads (Beckman Coulter, A63881) and prepared for sequencing using the Opto Plasma B Discovery Sanger Prep Kit (Supplemental Figure 12F) (Berkeley Lights, 750-02041). Nucleotide sequences were determined via Sanger sequencing in reverse orientation, and the resulting data were analyzed in Geneious Biologics using the single-clone antibody analysis pipeline for annotation. The paired heavy- and light-chain sequences of selected mAbs were connected via a flexible linker to form a single-chain variable fragment (scFv), which was then cloned into mammalian expression vectors (pcDNA 3.4 TOPO-6×his-tag; A14697). The scFv sequence of the Hu-B03 antibody is as follows: EVQLVESGGGLVKPGGSLRLSCAASGFTFSSYAMSWVRQAPGKGLEWVATISSIGFTYYPDSVKGRFTISRDNSKNTLYLQMSSLRAEDMAVYYCARPSYYGNYGWYFDVWGQGTLVTVSSGGGGSGGGGSGGGGSDIQMTQSPSSLSASVGDRVTISCRASQDIRNYLNWYQQKPGKAPKLLIYYTSRLHSGVPSRFSGSGSGTDYTLTISSLQPEDIATYYCQQGNTFPLTFGGGTKVEIK.

### Expression and prification of h/mCD155 scFv.

Expression vectors containing scFvs were transfected into HEK293F cells using the TA293 transfection reagent (Kairui Biotech, K20001) in KPM serum-free medium (Kairui Biotech, K03125). After a 7-day incubation, cell culture supernatants were harvested and purified using an Ni²^+^ affinity column (Cytiva, 17371202). The bound mAbs were equilibrated with a buffer containing 20 mM Na_2_HPO_4_ and 150 mM NaCl (pH 7.0), and eluted with a buffer containing 20 mM Na_2_HPO_4_, 150 mM NaCl, and 500 mM imidazole (pH 7.0) using an AKTA Pure 150 system (Cytiva). The eluted antibodies were then concentrated and resuspended in PBS using 30 kDa molecular weight cutoff centrifugal filter units (Millipore, UFC903024). The purified antibodies were validated by SDS-PAGE (Supplemental Figure 12G) and stored at –80°C.

### Construction of h/mCD155-CARs.

The full hCD155-CAR construct included a CD8 signal peptide, antigen-specific scFvs (B03 and Hu-B03), a CD8α hinge and transmembrane domain, the 4-1BB costimulatory domain, and the CD3ζ intracellular domain (Supplemental Figure 12H). Similarly, the mCD155-CAR construct consisted of a CD8 signal peptide, antigen-specific scFv (G06), a CD28 hinge and transmembrane domain, the CD28 costimulatory domain, and the CD3ζ intracellular domain (Supplemental Figure 12I). CAR expression was assessed by flow cytometry, staining CAR-T cells with biotinylated-hCD155 recombinant protein (for hCD155-CAR) (Supplemental Figure 12J) or mCD155-Fc recombinant protein (for mCD155-CAR) (Supplemental Figure 12K). All constructs were codon-optimized and cloned into the MMLV retroviral vector using commercial cloning services (VectorBuilder). The hCD19-CAR construct was purchased directly from VectorBuilder, using the same backbone as the h/mCD155-CAR. The mCD19-CAR construct was generated following standard protocols, using the 1D3 antibody sequence, as previously described (70).

Additional methods are presented in Supplemental data.

### Statistics.

Statistical analyses were performed using GraphPad Prism 9 (GraphPad Software), except for scRNA-Seq data, which were analyzed with R. Data are presented as mean ± SD, unless otherwise indicated. Comparisons between 2 independent or matched groups were conducted using 2-tailed Student’s *t* tests or paired *t* tests, respectively; comparisons among multiple matched or independent groups were performed using 1-way ANOVA with or without repeated measures. Interactions between 2 factors were assessed by 2-way ANOVA with (e.g., repeated measure over time) or without repeated measures. Anti-CD155 scFv dose-response curves were assessed using a 4-parameter logistic nonlinear regression model, from which EC_50_ values were calculated. Survival data were evaluated using the Kaplan-Meier method and log-rank tests. *P* values were adjusted for multiple comparisons by Tukey method. A *P* value of less than 0.05 was considered statistically significant. Detailed statistical results are provided in the figure legends.

### Study approval.

Human CD34^+^ HSPCs were isolated from cord blood purchased from StemCyte. AML specimens were obtained from patients diagnosed with AML at City of Hope National Medical Center (COHNMC) under a protocol approved by the COHNMC IRB (no. 18067). Healthy donor samples were collected following consent under IRB 06229. All patient sample acquisitions were approved by the COHNMC IRB in accordance with an assurance filed with the Department of Health and Human Services and adhered to the principles outlined in the Declaration of Helsinki. All animal care and experimental procedures followed federal or national guidelines and were approved by the IACUC at City of Hope or the Chongqing International Institute for Immunology.

### Data availability.

All data generated or analyzed during this study are included in this article and its supplemental information files. Values for all data points in graphs are reported in the Supporting Data Values file. Other data and materials are available upon reasonable request from the corresponding authors.

## Author contributions

TX, JY, MAC, and TZ conceived and designed the project. TX, GW, PY, Zhenlong Li, SL, RY, RM, and Zhiyao Li conducted experiments. TX, DL, JZ, XL, and JM analyzed and interpreted data. TX, JY, and MAC wrote, reviewed, and/or revised the manuscript. GM provided material support. JY, MAC, and TZ supervised the study and acquired funding. All authors discussed the results and commented on the manuscript.

## Supplementary Material

Supplemental data

Supplemental table 1

Supporting data values

## Version 1 06/06/2025

In-Press Preview

## Version 2 08/01/2025

Electronic publication
