# Supplementary material for: CAR-T cells targeting CD155 reduce tumor burden in preclinical models of leukemia and solid tumors
Source: J Clin Invest. 2025 Jun 6;135(15):e189920. doi: 10.1172/JCI189920 (PMC12321399; doi:10.1172/JCI189920)
Supplement: Supplemental data [file jci-135-189920-s205.pdf]

Supplemental Information

CAR-T cells targeting CD155 reduce tumor burden in preclinical models of leukemia and solid tumors

Authors

Tianchen Xiong[1,2], Ge Wang[1,2,3], Peng Yu[4], Zhenlong Li[1,2], Debao Li[4], Jianying Zhang[5], Song Lu[4], Ruiqi Yang[4], Xiaolong Lian[4], Jianhong Mi[4], Rui Ma[1,2], Zhiyao Li[1,2], Guido Marcucci[6], Tingting Zhao[4], Michael A. Caligiuri[1,2], Jianhua Yu[7,8,9]

Author affiliation

[1]Department of Hematology & Hematopoietic Cell Transplantation, City of Hope National Medical Center, Los Angeles, CA 91010, USA

[2]Hematologic Malignancies Research Institute, City of Hope National Medical Center, Los Angeles, CA 91010, USA

[3]Department of Neurology, Xiangya Hospital, Central South University, Changsha, 410008, China

[4]Chongqing International Institute for Immunology, Chongqing, 401338, China

[5]Department of Computational and Quantitative Medicine, City of Hope National Medical Center, Los Angeles, CA 91010, USA

[6]Gehr Family Center for Leukemia Research, Hematologic Malignancies Research Institute, City of Hope National Medical Center, Los Angeles, CA 91010, USA

[7]Division of Hematology & Oncology, Department of Medicine, School of Medicine, University of California, Irvine, CA 92697, USA

[8]Institute for Precision Cancer Therapeutics and Immuno-Oncology, Chao Family Comprehensive Cancer Center, University of California, Irvine, CA 92697, USA

[9]The Clemons Family Center for Transformative Cancer Research, University of California, Irvine, CA 92697, USA

Authorship note: T.X., G.W., P.Y. and Z.L. contributed equally to this work.

Correspondence authors: Jianhua Yu Ph.D., jianhuay@uci.edu; Michael A. Caligiuri, M.D. mcaligiuri@coh.org; Tingting Zhao Ph.D., zhaotingting@iiicq.vip.

Supplemental Methods

Retrovirus production

Retrovirus production was carried out using Lipofectamine™ 3000 reagent, following the manufacturer's protocol (Invitrogen). For hCD155-CAR and hCD19-CAR production, GP2-293 retroviral packaging cells were plated in 10-cm dishes and cultured for 48 hours. Before transfection, the medium was refreshed with 10 mL of prewarmed medium without disturbing the cells. For each transfection, 15 µg of plasmid DNA (10 µg of hCD155-CAR plasmid and 5 µg of RD114 envelope plasmid) was mixed with 470 µL of Opti-MEM™ medium and 30 µL of P3000™ reagent. Concurrently, 470 µL of Opti-MEM™ and 30 µL of Lipofectamine™ 3000 reagent were prepared in a separate tube. The diluted DNA mixture was then combined with the diluted Lipofectamine™ 3000 reagent dropwise with gentle vortexing. After a 15-minute incubation at room temperature, the transfection mixture was gently added to the GP2-293 cells. The medium was replaced with 10 mL of prewarmed medium 12 hours later. After an additional 36–72 hours of incubation, the virus-containing supernatant was collected and filtered through a 0.45-µm filter (Millipore, SLHVM33RS) to remove cell debris. The virus-containing supernatant was then aliquoted and stored at –80°C. For mCD155-CAR and mCD19-CAR production, HEK293T cells were used in conjunction with the pCL-Eco packaging plasmid instead of GP2-293 cells and the RD114 envelope plasmid.

Generation of h/mCD155 CAR-T cells and h/mCD19 CAR-T cells

For human T cell isolation, peripheral blood mononuclear cells (PBMCs) were separated by density gradient centrifugation, and T cells were purified using the RosetteSep™ Human T Cell Enrichment Cocktail (STEMCELL). Human T cells were then stimulated with CD3/CD28 T cell Activator Dynabeads (STEMCELL) and cultured in RPMI 1640 medium (Gibco) supplemented with 10% FBS (Gibco), 2 mM L-glutamine, 1% penicillin/streptomycin, 1% sodium pyruvate, 1% non-essential amino acids, 0.1% 2-mercaptoethanol (all from Gibco), along with recombinant human IL-2 (100 U/ml) and IL-15 (0.5 ng/ml), provided by the National Institutes of Health. For mouse T cell isolation, spleens from wild-type C57BL/6 mice were mechanically dissociated and filtered through a 70-µm strainer (Biologix). Red blood cells were lysed with ACK lysis buffer (2 mL per spleen) for 5 minutes at room temperature. Splenocytes were then washed with FACS buffer (DPBS containing 2% FBS and 1 mM EDTA, pH 8.0), and mouse T cells were purified

using the EasySep™ Mouse T Cell Isolation Kit (STEMCELL). Mouse T cells were stimulated with CD3/CD28 T cell Activator Dynabeads (Thermo Fisher Scientific) and cultured in RPMI 1640 medium with 10% FBS, 2 mM L-glutamine, 1% penicillin/streptomycin, 1% sodium pyruvate, 1% non-essential amino acids, 1% HEPES, and 0.1% 2-mercaptoethanol (all from Gibco), along with recombinant human IL-2 (100 U/ml) and IL-15 (0.5 ng/ml). Both human and mouse T cells were cultured at 37°C for 48 hours without disturbance. To generate h/mCD155 CAR-T cells, 24-well plates were coated with RetroNectin (20 μg/mL, Takara) at 4°C overnight. On day 2, the plates were blocked with PBS containing 2% bovine serum albumin for 30 minutes. Virus supernatant containing h/mCD155-CARs or h/mCD19-CARs was added to the wells (2 mL per well), and the plates were centrifuged at 2,000 g for 2 hours at 32°C. Activated human or mouse T cells (10^5 per well) were then added to the virus-enriched plates and cultured at 37°C for another 72 hours before assessing CAR transduction efficiency. The mock T cells used in this study are infected with empty vector, serving as negative controls with no CAR expression or antigen specificity.

CDR grafting and murine scFv humanization

The murine amino acid sequence of scFv B03 was analyzed using BIOVIA Discovery Studio Visualizer (DS Visualizer, version 19.1), with annotations for framework regions (FRs) and complementarity-determining regions (CDRs). The 3D structure of the antibody was modeled using templates for the FR and CDR regions, and the optimal model was selected based on sequence similarity and energy minimization. DS Visualizer identified three types of canonical structure-determining residues, and their roles in maintaining the antibody's conformation were confirmed through 3D visualization. Next, high-homology human FRs from the Fab sequence database were selected, and the murine CDRs were grafted onto these human FRs. Key residues for back-mutation were identified based on canonical structure-determining residues and their predicted impact on structural stability and binding affinity (1).

Antibody humanness analysis

The humanness of the humanized scFv was evaluated using the online Humanness (HScore) tool (https://www.bioinf.org.uk/abs/shab/) (2). The donor mouse IgG sequence and the acceptor human IgG sequence were used as negative and positive controls, respectively. The similarity distribution

of the humanized scFv, including both light and heavy chains, was then analyzed and quantified using Z-score values.

Flow cytometry

Cell surface staining was conducted at 4°C for 30 minutes in the dark. Briefly, cells were blocked using purified NA/LE human BD Fc Block™ and stained with the indicated antibodies at 4°C for 30 minutes. After staining, cells were washed and resuspended in ice-cold FACS buffer (DPBS containing 2% FBS and 1 mM EDTA, pH 8.0) for flow cytometric analysis. For cellular scFv binding assays, HEK293T cells overexpressing human or murine CD155 were harvested, washed with FACS buffer, and incubated with serial dilutions of 6×His-tagged scFvs for 1 hour at 4°C. After incubation, cells were washed and stained with anti-His-APC (BioLegend) at 4°C for 30 minutes before flow cytometry analysis. To detect hCD155-CAR expression, hCD155 CAR-T cells were incubated with biotinylated recombinant hCD155 protein (BioLegend), followed by secondary staining with streptavidin-APC (BD Biosciences). For mCD155-CAR detection, mCD155 CAR-T cells were incubated with recombinant mCD155-Fc protein (BioLegend) and then stained with anti-Fc-APC (BioLegend). For proliferation assessment, mock T cells or CD155 CAR-T cells were stained using the CellTrace Violet Cell Proliferation Kit (Thermo Fisher Scientific) according to the manufacturer's instructions. Proliferation was measured on day 3 post-staining by trace dilution. For intracellular cytokine staining, mock T cells or CD155 CAR-T cells were co-cultured with tumor cells in the presence of BD GolgiPlug™ (BD Biosciences) at 37°C for 4 hours. Cells were then surface-stained, followed by fixation and permeabilization using the Cytofix/Cytoperm Fixation/Permeabilization Solution Kit (BD Biosciences). Intracellular staining for TNF and IFNγ was performed according to the manufacturer's protocol. To assess tumor cell death, tumor cells were co-cultured with mock T cells or CD155 CAR-T cells for 4 hours, stained with APC-conjugated Annexin V and 4′,6-diamidino-2-phenylindole (DAPI) (BD Biosciences), and analyzed by flow cytometry. All samples were analyzed using a Fortessa X-20 with FACSDiva Software (BD Biosciences), and data analysis was performed with FlowJo 10.8.1 (Tree Star). Gate margins were determined by isotype controls and fluorescence minus one (FMO) controls.

Antibodies and reagents for flow cytometry

The following antibodies and reagents were purchased from BioLegend: hCD155 (SKII.4, 337617), biotinylated human CD155 recombinant protein (796604), anti-His tag antibody

(J095G46, 362605), hCD3 (UCHT1, 300440), hCD4 (RPA-T4, 300521), hCD8 (HIT8a, 300926), hCD62L (DREG-56, 304823), hCD45RA (HI100, 304136), Annexin V (640912), hIFNγ (B27, 506507), hCD19 (HIB19, 302206), hCD14 (63D3, 367124), hCD11c (3.9, 301643), hCD66b (6/40c, 392903), h/mCD11b (M1/70, 101206), streptavidin (405226), hCD69 (FN50, 310922), hCD25 (BC96, 302610), hCD10 (HI10a, 312237), hCD90 (5E10, 328125), recombinant mouse CD155-Fc chimera (786604), anti-human IgG Fc antibody (M1310G05, 410712), mCD155 (TX56, 131529), mCD3ε (145-2C11, 100320), mTER-119 (TER-119, 116222), mCD19 (6D5, 115538), mCD3 (17A2, 100218), mNK1.1 (PK136, 108706), mLy-6C (HK1.4, 128036), mLy-6G (1A8, 127643), mF4/80 (QA17A29, 157304), mCD11c (N418, 117353) and mI-A/I-E (M5/114.15.2, 107641). The following antibodies and reagents were purchased from BD Biosciences: Cytofix/Cytoperm Fixation/Permeabilization Solution Kit (555028), BD GolgiPlug™ (555029), purified NA/LE human BD Fc Block™ (564765), hHLA-DR (G46-6, 560652), hCD56 (NCAM16.2, 563169), hTNF (MAb11, 563418), hCD34 (581, 555822), hCD38 (HIT2, 560981), hCD3 (UCHT1, 563423), hCD45 (HI30, 563792), hCD7 (M-T701, 555360), mLy-6A/E (D7, 565507), h/mCD11b (M1/70, 552850), mLy-6G and Ly-6C (RB6-8C5, 565033), mB220 (RA3-6B2, 552772), mCD16/32 (2.4G2, 563006), mSca-1 (D7, 565507), mCD34 (RAM34, 560518), streptavidin (554067), and DAPI (564907). The CellTrace™ Violet Cell Proliferation Kit (C34571), hFLT3 (BV10A4H2, 12-1357-42), mc-Kit (ACK2, 67-1172-82), and mCD117 antibody (ACK2, 67-1172-82) were purchased from Thermo Fisher Scientific. Human TIGIT Protein, Fc Tag (MALS verified) (TIT-H5254-100ug) and Human DNAM-1/CD226 Protein, Fc Tag (MALS verified) (DN1-H5257-100ug) were purchased from Acro Biosystems.

Luminescence-based *in vitro* cytotoxicity assays

Mock T cells or CD155 CAR-T cells were co-cultured with firefly luciferase-labeled MOLM13, U937, or THP-1 cells at the indicated E:T ratios. Target cells alone were plated at the same cell density to determine maximal luciferase expression, measured as relative light units (RLU). After 4 hours of co-culture, 100 μL of the cell mixture was transferred to a 96-well white luminometer plate. Subsequently, 10 μL of luciferase substrate (Promega) was added to each well, and luminescence was detected using a plate reader. Cell viability was calculated as a percentage based on the luciferase activity in wells with target cells alone, using the formula: % cell viability =

[(RLU from wells with effector and target cell co-culture) / (RLU from wells with target cells only)] × 100.

In vitro PBMC killing assays

A total of 5×10^6 donor-matched PBMCs were co-cultured with 1×10^6 CellTrace Violet (CTV)-labeled CD19 CAR-T cells or Hu-B03 CAR-T cells for 12 hours. Cytotoxicity was then assessed by flow cytometry to evaluate cell death across immune cell subsets.

Representative real-time cell analysis (RTCA)-based *in vitro* killing assays

To perform RTCA-based assays, 50 μL of cell culture medium was added to each well of an E-plate (Cat#300601010, Agilent), a 96-well plate with a glass bottom coated with gold microelectrodes covering approximately 75% of the well area. The E-plate was connected to the system to ensure proper electrical contact and to obtain background impedance readings in the absence of cells. A549 and Capan-1 cells were used as target cells, with 5,000 cells in 50 μL of media plated into each well of the E-plate. The target cells were cultured overnight in the RTCA system housed within a CO_2 incubator. The following day, 100 μL of media containing mock T cells or CD155 CAR-T cells was added to the E-plate, and co-cultures were maintained in the RTCA system for at least an additional 60 hours. The growth and cell index of target cells were continuously monitored and measured according to the manufacturer's instructions.

Animal experiments

For the human AML cell engraftment experiment, 6–8-week-old NSG mice were transplanted with 1×10^5 MOLM13 or U937 cells via tail vein injection. On day 7 after tumor implantation, the mice received an intravenous (i.v.) injection of 2×10^6 mock T cells, CD155 CAR-T cells, or PBS. For the human lung cancer cell implantation experiment, 1×10^5 A549 cells were transplanted into 6–8-week-old NSG mice via i.v. injection. On day 5 after tumor implantation, the mice were injected i.v. with 2×10^6 mock T cells or CD155 CAR-T cells. For the human pancreatic cancer cell implantation experiment, 1×10^5 Capan-1 cells were injected intraperitoneally (i.p.) into 6–8-week-old NSG mice. On day 5 post-tumor implantation, mice received an i.v. injection of 2×10^6 mock T cells or CD155 CAR-T cells. For the murine pancreatic cancer cell implantation experiment, 1×10^6 KPC cells were i.p. injected into 6–8-week-old C57BL/6J mice. On day 7 post-tumor implantation, mice received an i.p. injection of 2×10^6 mock T cells or mCD155 CAR-

T cells. For the *in vivo* PBMC killing assay, 1×10^7 donor-matched PBMCs were i.v. injected into 6–8-week-old NSG mice along with 2×10^6 mock T cells or Hu-B03 CAR-T cells. On day 7 post-injection, bone marrow was collected for hematologic toxicity analysis. For the *in vivo* $CD34^+$ HSPCs differentiation experiment, a total of 1×10^5 $CD34^+$ HSPCs were transplanted into 6–8-week-old NSG-SGM3 mice concurrently with the indicated number of either mock T cells or Hu-B03 CAR-T cells. Mice were sacrificed on day 30 post-injection for analysis of human $CD34^+$ HSPCs and their differentiation, including mature lymphoid and myeloid populations in the bone marrow. All mice were monitored regularly for AML, lung, or pancreatic cancer progression, and tumor growth was imaged at various time points. Mice showing signs of distress, discomfort, pain, lethargy, inability to groom or access food/water, or experiencing 20% weight loss compared to age-matched controls were euthanized via CO_2 inhalation. Tumor burden was assessed using *in vivo* bioluminescence imaging via the IVIS Imaging System at the City of Hope Small Animal Imaging Core. For luciferase detection, 200 µL of 15 mg/mL D-luciferin (Caliper Life Sciences) in PBS was injected intraperitoneally before imaging. The group allocation was blinded to the observers. For the *in vivo* safety experiment, 2×10^6 mock T cells or mCD155 CAR-T cells were injected i.v. into 6–8-week-old C57BL/6J mice. Mice were monitored for weight and survival throughout the study. On day 30 post-injection, spleens and bone marrow was collected for hematologic toxicity analysis, and blood samples along with various organs were harvested for cytokine assessment and H&E staining. The H&E staining results were analyzed in a blinded fashion by a mouse pathologist.

Assessment of cytokine levels in sera

Blood was collected from mice via tail clipping and allowed to clot for 60 minutes at room temperature. The clotted blood was then centrifuged at 1,500 g for 10 minutes at 4°C. Serum was collected, and cytokine levels were measured using assays provided by RayBiotech Life Inc (QAM-IFN-1).

Histology

For the murine safety study, tissues were collected from experimental mice and fixed in 10% neutral buffered formalin for up to 72 hours, followed by paraffin embedding and sectioning at 5 µm thickness. Tissue sections were stained with hematoxylin and eosin (H&E) or with anti-mCD155 antibodies (clone 305, Invitrogen, MA5-29762; or clone G06) using standard protocols

at the City of Hope Pathology Core Laboratory. For neurotoxicity evaluation, brain sections were stained with anti-IBA1 and GFAP antibodies, also processed by the same core facility. For human immunohistochemistry (IHC), tissue microarray (TMA) slides containing normal and cancerous tissues were provided by the City of Hope Pathology Core Laboratory and stained with anti-hCD155 antibody (D8A5G, 81254S, CST) according to standard procedures. All stained slides were mounted, digitally scanned, and analyzed using NDP.view2 software (Hamamatsu Photonics).

Cross-organ off-target transcriptomic atlas (COOTA) (3)

To evaluate on-target, off-tumor effects, we analyzed and combined 49 single-cell RNA sequencing (scRNA-seq) datasets across 9 healthy tissues (Supplemental Table 1). The R package Seurat (version 4.2.0) was used for scRNA-seq data analysis. Stringent quality control was performed to filter out low-quality cells based on three metrics: total UMI counts, the number of detected genes, and the proportion of mitochondrial gene UMI counts. We then carried out the following steps: 1. Normalization: The NormalizeData function was used for library size correction and logarithmic transformation. 2. Variable Feature Selection: The FindVariableFeatures function selected the top 2,000 highly variable genes (HVGs). 3. Scaling: The ScaleData function was applied to scale the HVG expression matrix. 4. Principal Component Analysis (PCA): RunPCA was used to perform PCA on the scaled HVG expression matrix, retaining the top 30 components for further analysis. 5. Dimensionality Reduction: The RunUMAP function was used for dimensionality reduction, embedding cells in coordinates for visualization. Clusters were annotated based on classical cell marker expression, identifying the main cell types such as T cells, B cells, NK cells, and epithelial cells. A single-gene count matrix was created and used to plot mean expression values across cell types. All preprocessing and analysis steps were run in Python 3.9 using RStudio v4.2.0, with the pheatmap function used for visualization.

Supplemental References

1. Wang Y, et al. Comparison of "framework Shuffling" and "CDR Grafting" in humanization of a PD-1 murine antibody. *Front Immunol.* 2024;15:1395854.
2. Abhinandan KR, and Martin AC. Analyzing the "degree of humanness" of antibody sequences. *J Mol Biol.* 2007;369(3):852-62.
3. Gottschlich A, et al. Single-cell transcriptomic atlas-guided development of CAR-T cells for the treatment of acute myeloid leukemia. *Nat Biotechnol.* 2023;41(11):1618-32.

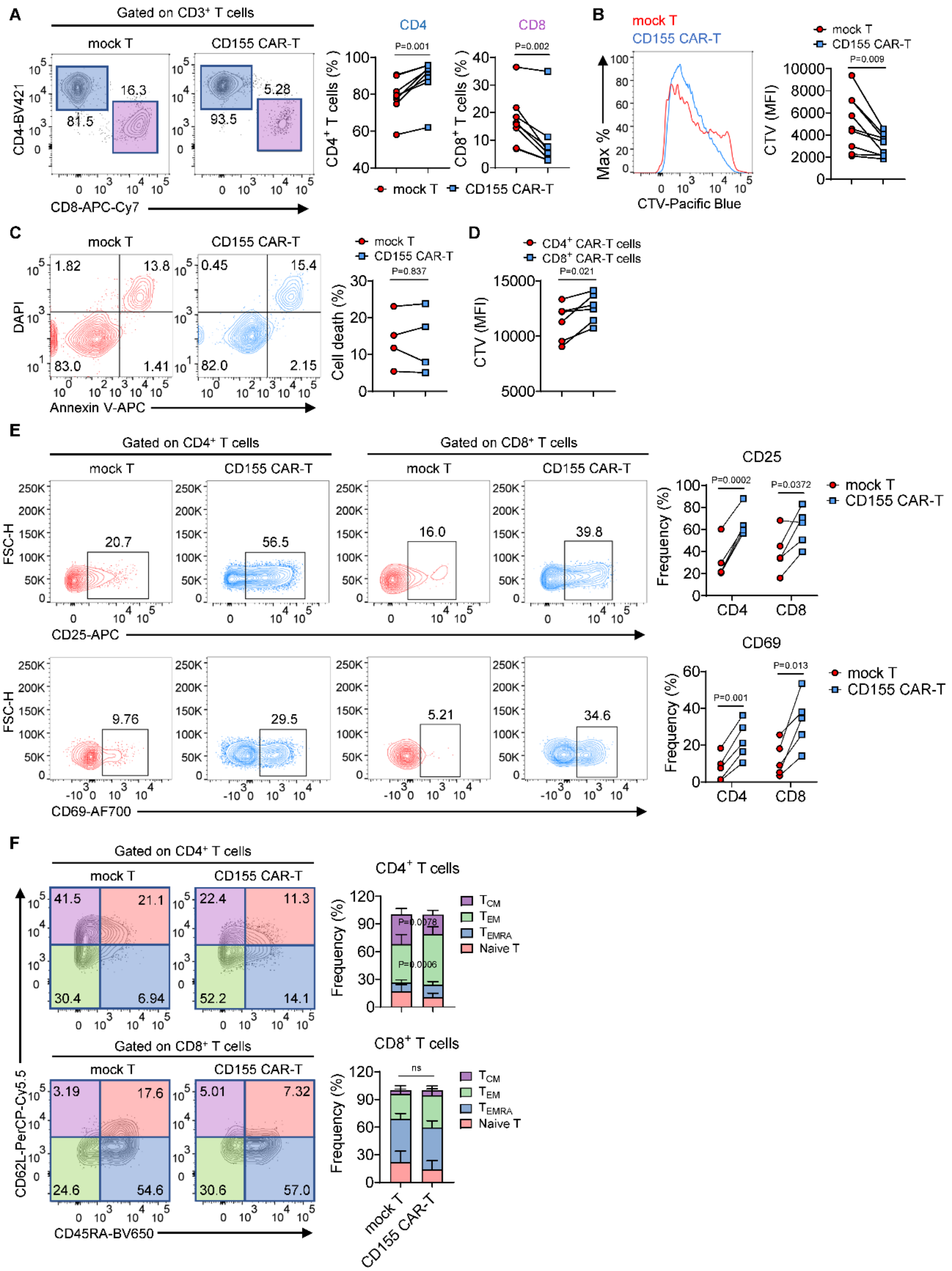

A
Gated on CD3⁺ T cells
mock T
CD155 CAR-T
CD4-BV421
CD8-APC-Cy7
16.3
81.5
5.28
93.5
CD4
CD8
P=0.001
P=0.002
CD4⁺ T cells (%)
CD8⁺ T cells (%)
mock T
CD155 CAR-T
B
mock T
CD155 CAR-T
Max %
CTV-Pacific Blue
P=0.009
CTV (MFI)
C
mock T
CD155 CAR-T
DAPI
Annexin V-APC
1.82
13.8
83.0
1.41
0.45
15.4
82.0
2.15
P=0.837
Cell death (%)
D
CD4⁺ CAR-T cells
CD8⁺ CAR-T cells
P=0.021
CTV (MFI)
E
Gated on CD4⁺ T cells
Gated on CD8⁺ T cells
FSC-H
CD25-APC
20.7
56.5
16.0
39.8
CD25
P=0.0002
P=0.0372
Frequency (%)
CD4
CD8
CD69-AF700
9.76
29.5
5.21
34.6
CD69
P=0.001
P=0.013
F
Gated on CD4⁺ T cells
41.5
21.1
30.4
6.94
22.4
11.3
52.2
14.1
CD4⁺ T cells
P=0.0078
P=0.0006
T_CM
T_EM
T_EMRA
Naive T
Gated on CD8⁺ T cells
3.19
17.6
24.6
54.6
5.01
7.32
30.6
57.0
CD8⁺ T cells
ns
CD62L-PerCP-Cy5.5
CD45RA-BV650

Supplemental Figure 1 Phenotypic and functional assessment of CD155 CAR-T cells. (**A**) Representative flow cytometry plots (left) and statistics (right) showing the percentage of $CD4^+$ and $CD8^+$ T cells in mock-transduced and CD155 CAR-T cells on day 10 after transduction (n = 8 individual donors). (**B**) Representative histograms and statistics showing the mean fluorescence intensity (MFI) of CellTrace Violet (CTV) in mock-transduced and CD155 CAR-T cells on day 10 after transduction (n = 9 individual donors). (**C**) Representative histograms and statistics showing the percentage of cell death of mock-transduced and CD155 CAR-T cells on day 10 after transduction, as measured by Annexin V and DAPI staining (n = 4 individual donors). (**D**) Statistics showing the MFI of CTV in $CD4^+$ and $CD8^+$ T cells from CD155-CAR positive cells on day 10 after transduction (n = 6 individual donors). (**E**) Representative flow cytometry plots (left) and statistics (right) showing the percentage of $CD25^+$ and $CD69^+$ T cells in mock-transduced and CD155 CAR-T cells on day 10 after transduction (n = 5 individual donors). (**F**) Representative flow cytometry plots (left) and statistics (right) showing the percentage of $CD45RA^+$ and $CD62L^+$ T cells in mock-transduced and CD155 CAR-T cells on day 10 after transduction (n = 8 individual donors). Naïve: $CD45RA^+$ $CD62L^+$, Central Memory (CM): $CD45RA^-$ $CD62L^+$, Effector Memory (EM): $CD45RA^-$ $CD62L^-$, Effector memory cells re-expressing CD45RA T cells (EMRA): $CD45RA^+$ $CD62L^-$. Data are shown as individual data points in dot plots (**A**-**E**) or represent the mean ± S.D. (**F**) and were analyzed by paired two-tailed Student's *t*-tests (**A**-**E**) or two-way ANOVA with repeated measures (**F**).

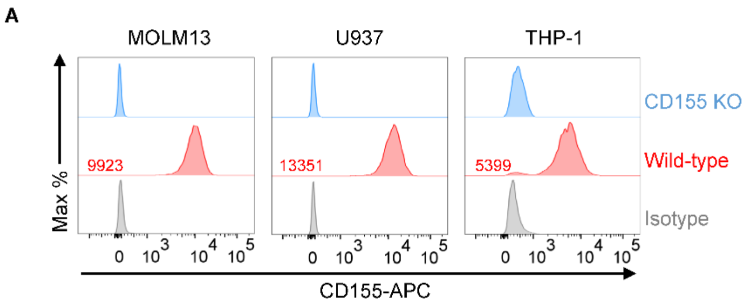

Supplemental Figure 2 Expression of CD155 on wild-type and CD155-KO AML cell lines. (**A**) Representative histograms showing the expression of CD155 on wild-type and CD155-KO AML cell lines.

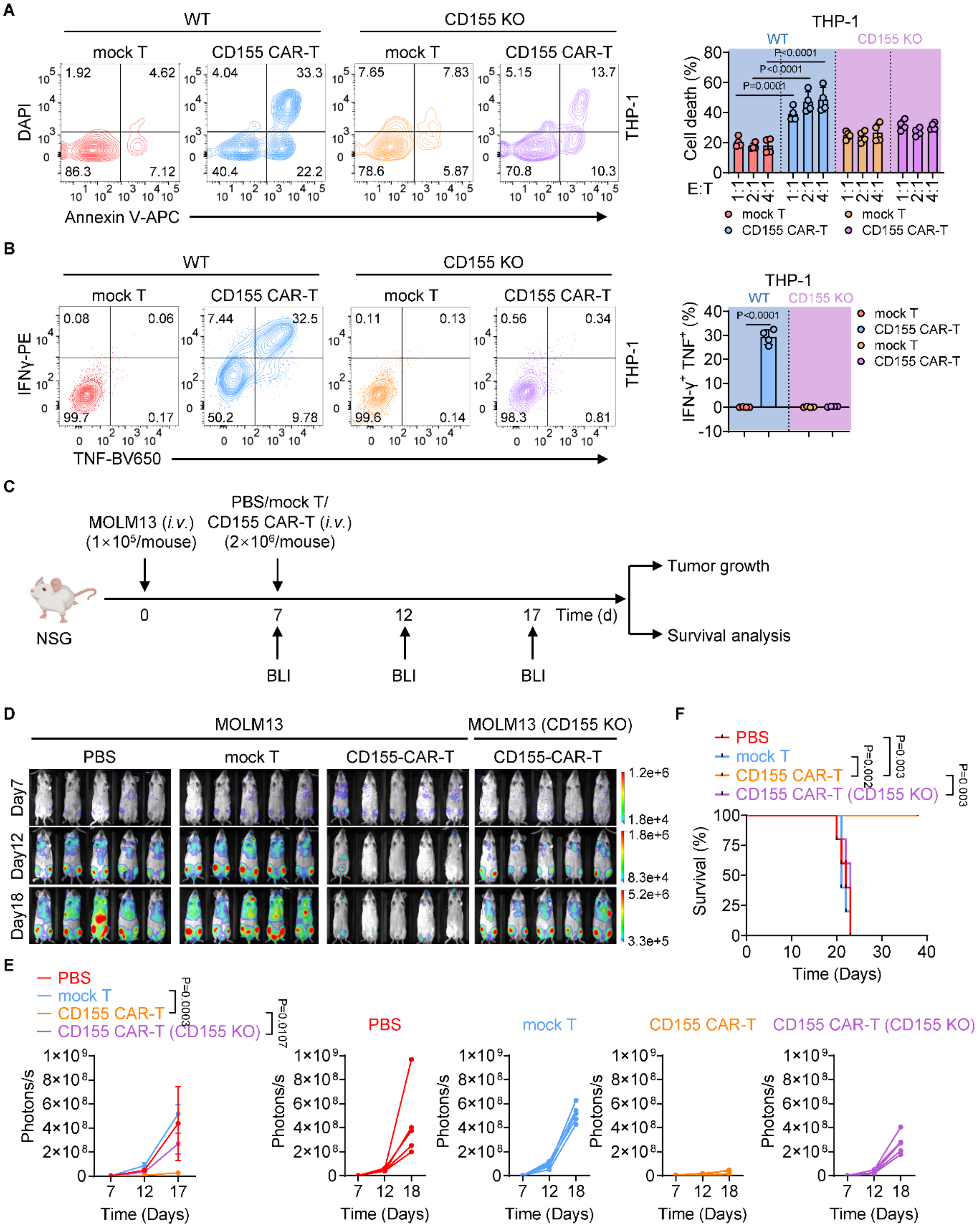

A
WT
CD155 KO
mock T
CD155 CAR-T
DAPI
Annexin V-APC
THP-1
Cell death (%)
E:T
B
IFNγ-PE
TNF-BV650
IFN-γ+ TNF+ (%)
C
MOLM13 (i.v.)
(1×10⁵/mouse)
PBS/mock T/
CD155 CAR-T (i.v.)
(2×10⁶/mouse)
NSG
Time (d)
BLI
Tumor growth
Survival analysis
D
MOLM13
MOLM13 (CD155 KO)
PBS
CD155-CAR-T
Day7
Day12
Day18
E
CD155 CAR-T (CD155 KO)
Photons/s
Time (Days)
F
Survival (%)

Supplemental Figure 3 CD155 CAR-T cells exhibit potent anti-tumor efficacy against AML cell lines *in vitro* and *in vivo*. (**A**) Representative flow cytometry plots (left) and statistics (right) showing the percentage of wild-type or CD155-KO THP-1 cell death co-cultured at indicated ratios with mock T or CD155 CAR-T cells for 4 hours (n = 4 individual donors). (**B**) Representative flow cytometry plots (left) and statistics (right) showing the percentage of $IFN\gamma^{+}$ and TNF^{+} T cells in mock T or CD155 CAR-T cells co-cultured with wild-type or CD155-KO THP-1 cells for 4 hours (n = 4 individual donors). (**C**) Diagram of the treatment scheme used for *in vivo* experiments. 1×10^5 wild-type or CD155-KO MOLM13 cells were i.v. injected into NSG mice, followed by an i.v. infusion of indicated number of mock T cells, CD155 CAR-T cells or PBS (n = 5 mice per group) on day 7. (**D-F**), Bioluminescence (BLI) images (**D**), quantification of tumor burden (**E**) and survival curves (**F**) in MOLM13 tumor-bearing mice after different treatments. Data represent the mean ± S.D. and were analyzed by one-way ANOVA with repeated measures (**B**) or two-way ANOVA with repeated measures (**A** and **E**). For Kaplan-Meier survival curves, statistical significance was calculated with a log-rank test (**F**).

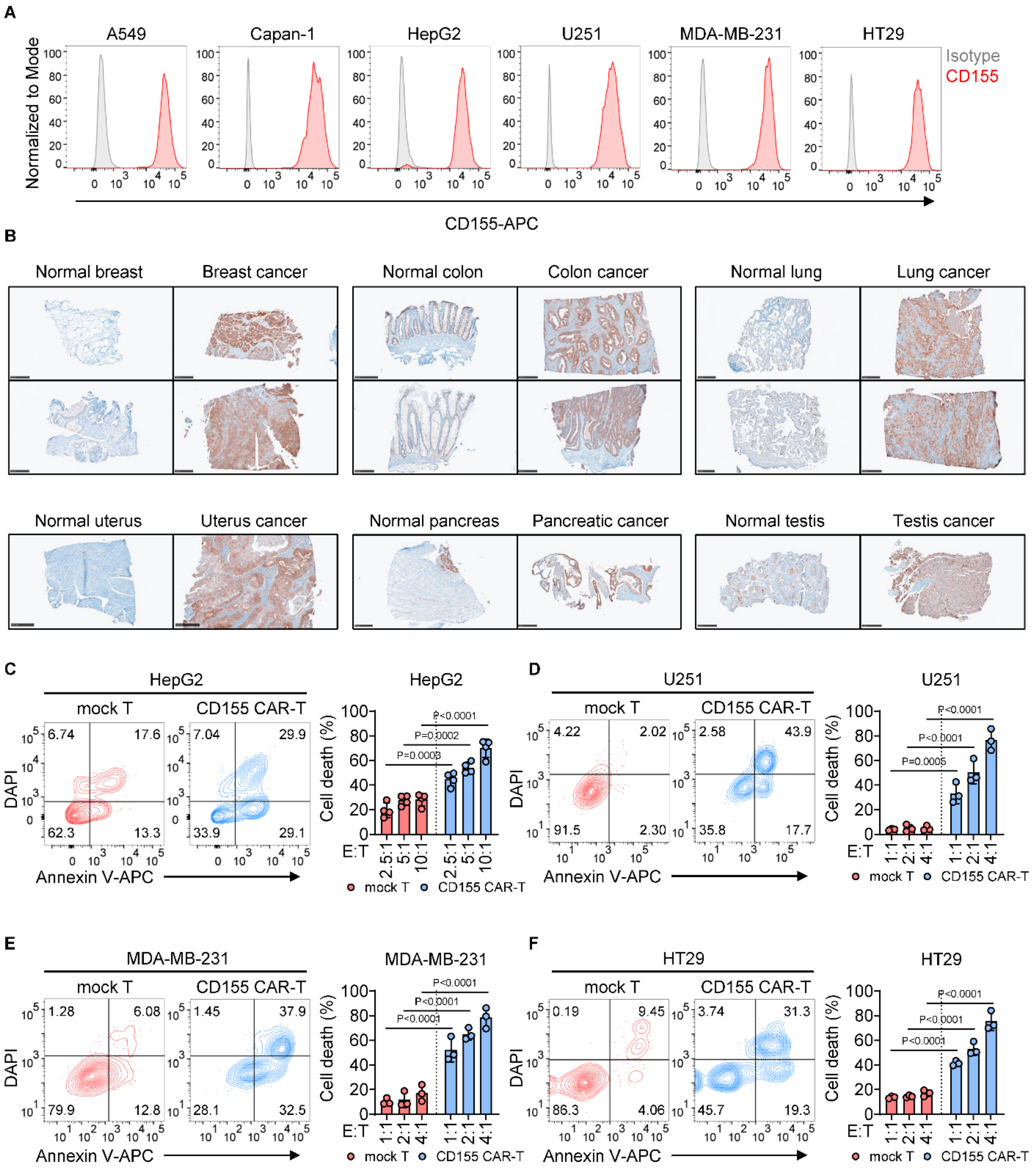

A
A549
Capan-1
HepG2
U251
MDA-MB-231
HT29
Normalized to Mode
Isotype
CD155
CD155-APC
B
Normal breast
Breast cancer
Normal colon
Colon cancer
Normal lung
Lung cancer
Normal uterus
Uterus cancer
Normal pancreas
Pancreatic cancer
Normal testis
Testis cancer
C
HepG2
mock T
CD155 CAR-T
DAPI
Annexin V-APC
6.74
17.6
62.3
13.3
7.04
29.9
33.9
29.1
Cell death (%)
P<0.0001
P=0.0002
P=0.0003
E:T
2.5:1
5:1
10:1
mock T
CD155 CAR-T
D
U251
4.22
2.02
91.5
2.30
2.58
43.9
35.8
17.7
P<0.0001
P<0.0001
P=0.0005
1:1
2:1
4:1
E
MDA-MB-231
1.28
6.08
79.9
12.8
1.45
37.9
28.1
32.5
P<0.0001
P<0.0001
P<0.0001
F
HT29
0.19
9.45
86.3
4.06
3.74
31.3
45.7
19.3
P<0.0001
P<0.0001
P<0.0001

Supplemental Figure 4 CD155 CAR-T cells display robust anti-tumor activity against solid tumors. (**A**) Representative histograms showing the expression of CD155 on solid tumor cell lines. (**B**) Immunohistochemical (IHC) analysis of hCD155 expression on normal and cancerous tissues, including breast, colon, lung, uterus, pancreas and testis. Scale bars represent 250 μm. (**C-F**) Representative flow cytometry plots (left) and statistics (right) showing the percentage of HepG2 (**C**), U251 (**D**), MDA-MB-231 (**E**) and HT29 (**F**) cell death co-cultured at indicated ratios with mock T or CD155 CAR-T cells for 4 hours (n = 4 individual donors in (**C**); n = 3 individual donors in (**D-F**). Data represent the mean ± S.D. and were analyzed by two-way ANOVA with repeated measures (**C-F**).

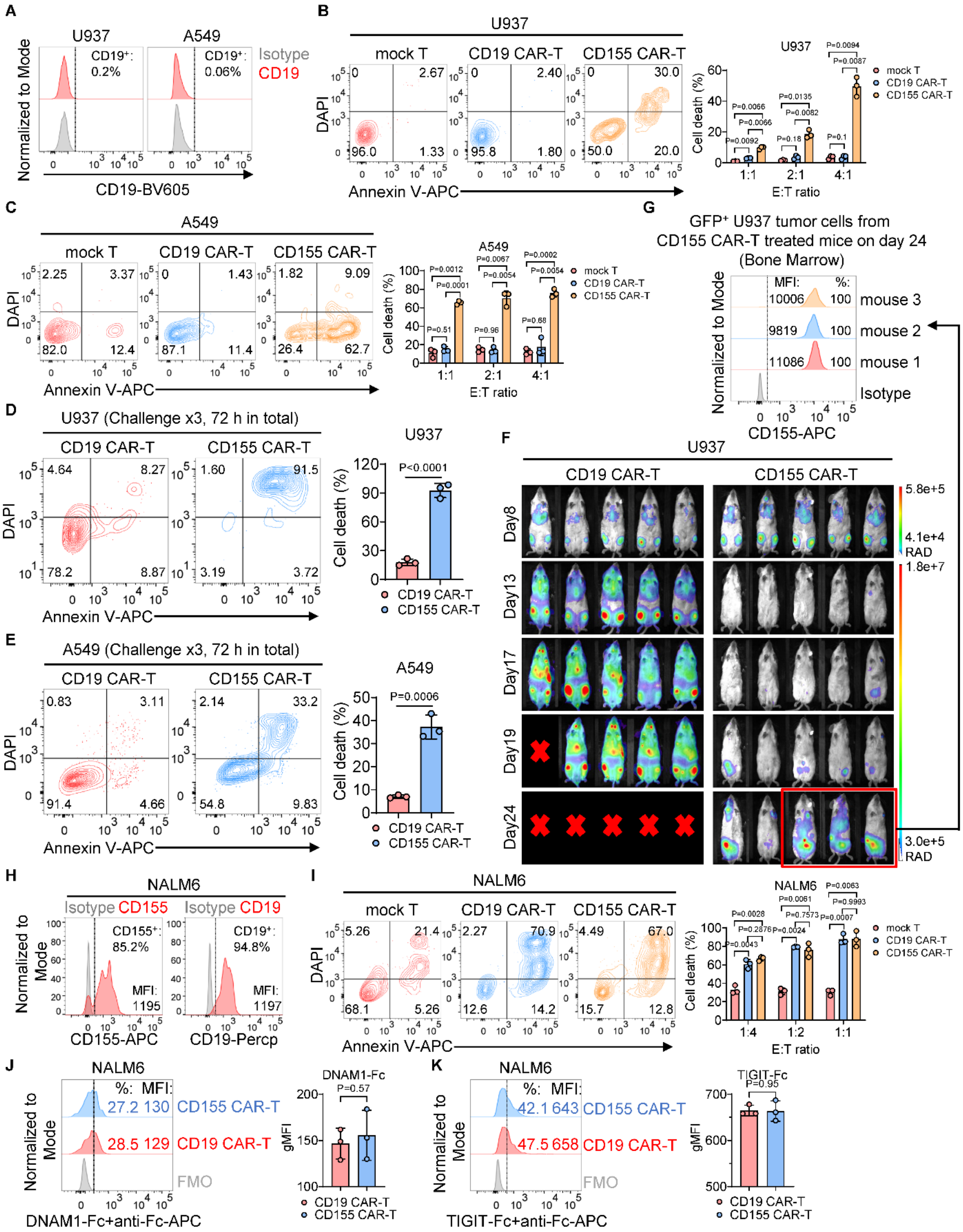
A
U937
A549
CD19⁺: 0.2%
CD19⁺: 0.06%
Isotype
CD19
Normalized to Mode
CD19-BV605
B
U937
mock T
CD19 CAR-T
CD155 CAR-T
2.67
96.0
1.33
2.40
95.8
1.80
30.0
50.0
20.0
DAPI
Annexin V-APC
Cell death (%)
E:T ratio
mock T
CD19 CAR-T
CD155 CAR-T
C
A549
mock T
CD19 CAR-T
CD155 CAR-T
2.25
3.37
82.0
12.4
1.43
87.1
11.4
1.82
9.09
26.4
62.7
DAPI
Annexin V-APC
Cell death (%)
E:T ratio
G
GFP⁺ U937 tumor cells from CD155 CAR-T treated mice on day 24 (Bone Marrow)
MFI:
%:
10006
100
mouse 3
9819
100
mouse 2
11086
100
mouse 1
Isotype
Normalized to Mode
CD155-APC
D
U937 (Challenge x3, 72 h in total)
CD19 CAR-T
CD155 CAR-T
4.64
8.27
78.2
8.87
1.60
91.5
3.19
3.72
DAPI
Annexin V-APC
U937
P<0.0001
Cell death (%)
CD19 CAR-T
CD155 CAR-T
E
A549 (Challenge x3, 72 h in total)
CD19 CAR-T
CD155 CAR-T
0.83
3.11
91.4
4.66
2.14
33.2
54.8
9.83
DAPI
Annexin V-APC
A549
P=0.0006
Cell death (%)
CD19 CAR-T
CD155 CAR-T
F
U937
CD19 CAR-T
CD155 CAR-T
Day8
Day13
Day17
Day19
Day24
5.8e+5
4.1e+4
RAD
1.8e+7
3.0e+5
RAD
H
NALM6
Isotype CD155
Isotype CD19
CD155⁺: 85.2%
MFI: 1195
CD19⁺: 94.8%
MFI: 1197
Normalized to Mode
CD155-APC
CD19-Percp
I
NALM6
mock T
CD19 CAR-T
CD155 CAR-T
5.26
21.4
68.1
5.26
2.27
70.9
12.6
14.2
4.49
67.0
15.7
12.8
DAPI
Annexin V-APC
NALM6
Cell death (%)
E:T ratio
mock T
CD19 CAR-T
CD155 CAR-T
J
NALM6
%: MFI:
27.2 130 CD155 CAR-T
28.5 129 CD19 CAR-T
FMO
Normalized to Mode
DNAM1-Fc+anti-Fc-APC
DNAM1-Fc
P=0.57
gMFI
CD19 CAR-T
CD155 CAR-T
K
NALM6
%: MFI:
42.1 643 CD155 CAR-T
47.5 658 CD19 CAR-T
FMO
Normalized to Mode
TIGIT-Fc+anti-Fc-APC
TIGIT-Fc
P=0.95
gMFI
CD19 CAR-T
CD155 CAR-T

Supplemental Figure 5 Comparative specificity and functional durability of CD155 CAR-T cells. (**A**) Representative histograms showing the percentage of CD19 positive cells on U937 and A549 cell lines. (**B** and **C**) Representative flow cytometry plots (left) and statistics (right) showing the percentage of U937 (**B**) and A549 (**C**) cell death co-cultured at indicated ratios with CD19 CAR-T or CD155 CAR-T cells for 4 hours (n = 3 individual donors). (**D** and **E**) CD19 CAR-T and CD155 CAR-T cells were co-cultured with U937 (**D**) and A549 (**E**) and subjected to three sequential challenges over a 72-hour period. Representative flow cytometry plots (left) and quantification (right) showing the percentage of tumor cell death co-cultured with CD19 CAR-T or CD155 CAR-T cells. (**F**) NSG mice were intravenously injected with 1×10^5 U937 tumor cells on day 0. On day 8 post-injection, mice received an intravenous infusion of 2×10^6 CD19 CAR-T cells or CD155 CAR-T cells (n = 5 per group). Tumor burden was monitored by bioluminescence imaging (BLI) at the indicated time points. (**G**) On day 24 post tumor injection, residual U937 tumor cells were isolated from CD155 CAR-T-treated mice and analyzed by flow cytometry to assess CD155 surface expression. (**H**) Representative histograms showing the MFI of CD155/CD19 and the percentage of CD155/CD19 positive cells on NALM6. (**I**) Representative flow cytometry plots (left) and quantification (right) showing the percentage of tumor cell death co-cultured with CD19 CAR-T or CD155 CAR-T cells. (**J** and **K**) Representative histograms (left) and quantification of MFI (right) showing the DNAM1-Fc (**J**) or TIGIT-Fc (**K**) binding to NALM6 cells in the presence of CD19 CAR-T cells or CD155 CAR-T cells. Data represent the mean ± S.D. (**B-E**, **I-K**) and were analyzed by paired two-tailed Student's *t*-tests (**D**, **E**, **J** and **K**) or two-way ANOVA with repeated measures (**B**, **C and I**).

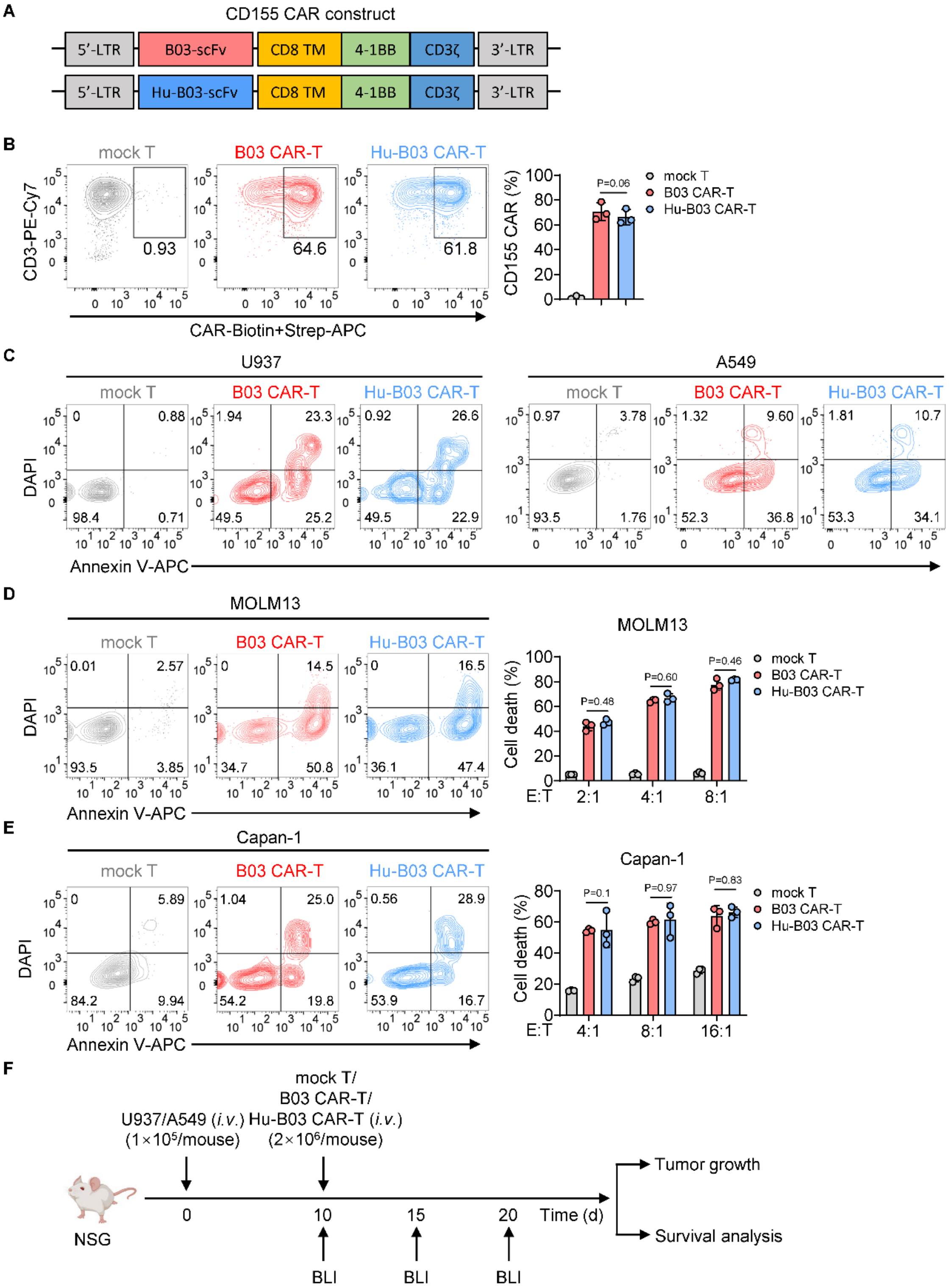
A
CD155 CAR construct
5'-LTR B03-scFv CD8 TM 4-1BB CD3ζ 3'-LTR
5'-LTR Hu-B03-scFv CD8 TM 4-1BB CD3ζ 3'-LTR
B
mock T
B03 CAR-T
Hu-B03 CAR-T
CD3-PE-Cy7
0.93
64.6
61.8
CAR-Biotin+Strep-APC
CD155 CAR (%)
P=0.06
mock T
B03 CAR-T
Hu-B03 CAR-T
C
U937
A549
DAPI
Annexin V-APC
D
MOLM13
Cell death (%)
P=0.48
P=0.60
P=0.46
E:T 2:1 4:1 8:1
E
Capan-1
P=0.1
P=0.97
P=0.83
E:T 4:1 8:1 16:1
F
mock T/
B03 CAR-T/
U937/A549 (i.v.) Hu-B03 CAR-T (i.v.)
(1×10⁵/mouse) (2×10⁶/mouse)
NSG
0 10 15 20 Time (d)
BLI BLI BLI
Tumor growth
Survival analysis

Supplemental Figure 6 Functional analysis of humanized CD155 CAR-T cells. (**A**) Schematic structure of the B03-CAR vector and the Hu-B03-CAR vector. (**B**) Representative flow cytometry plots (left) and statistics (right) showing the percentage of CD155-CAR positive T cells on day 5 after transduction, analyzed using flow cytometry by staining CAR-T cells with biotinylated-hCD155 recombinant protein and strep-APC (n = 3 individual donors). (**C**) Representative flow cytometry plots showing the percentage of U937 (left) or A549 (right) cell death co-cultured at indicated ratios with mock T, B03 CAR-T or Hu-B03 CAR-T cells for 4 hours (n = 3 individual donors). (**D** and **E**) Representative flow cytometry plots (left) and statistics (right) showing the percentage of MOLM13 (**D**) or Capan-1 (**E**) cell death co-cultured at indicated ratios with mock T, B03 CAR-T or Hu-B03 CAR-T cells (n = 3 individual donors). (**F**) Diagram of the treatment scheme used for *in vivo* experiments. 1×10^5 U937 or A549 tumor cells were i.v. injected into NSG mice, followed by an i.v. infusion of indicated number of mock T, B03 CAR-T or Hu-B03 CAR-T cells. Data represent the mean ± S.D. and were analyzed by one-way ANOVA (**B**) or two-way ANOVA with repeated measures (**D** and **E**).

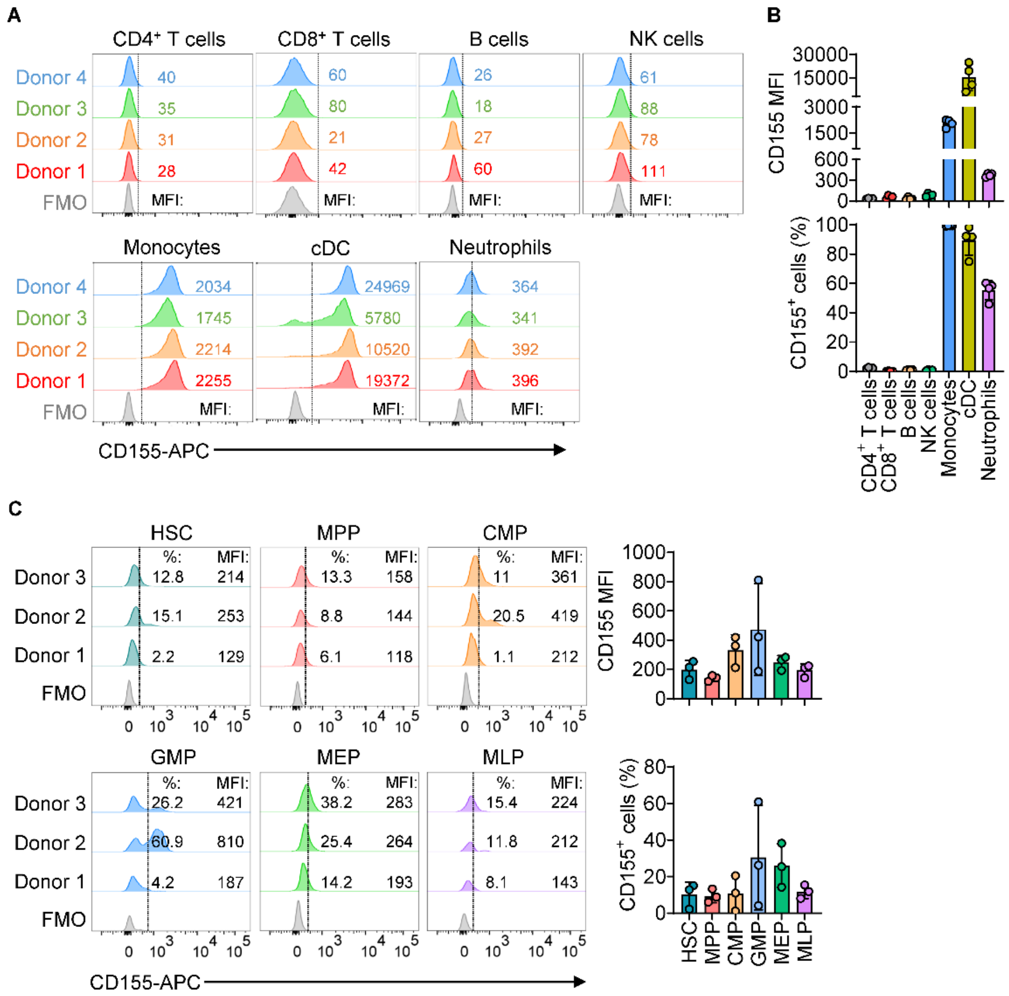

Supplemental Figure 7 Assessment of CD155 expression across human hematopoietic compartments. (**A** and **B**) Representative histograms (**A**) and statistics (**B**) showing the MFI of CD155 (upper in **B**) and the percentage of CD155 positive cells (lower in **B**) across various cell subsets of PBMCs from healthy donors (n = 4 individual donors). (**C**) Representative histograms and statistics showing the MFI of CD155 (upper right) and the percentage of CD155 positive cells (lower right) across various cell subsets of PBMCs from healthy donors (n = 4 individual donors). HSC: hematopoietic stem cell, MPP: multipotent progenitor, CMP: common myeloid progenitor, GMP: granulocyte-macrophage progenitor, MEP: megakaryocyte-erythroid progenitor, MLP: multilymphoid progenitor. Data represent the mean ± S.D. (**B** and **C**).

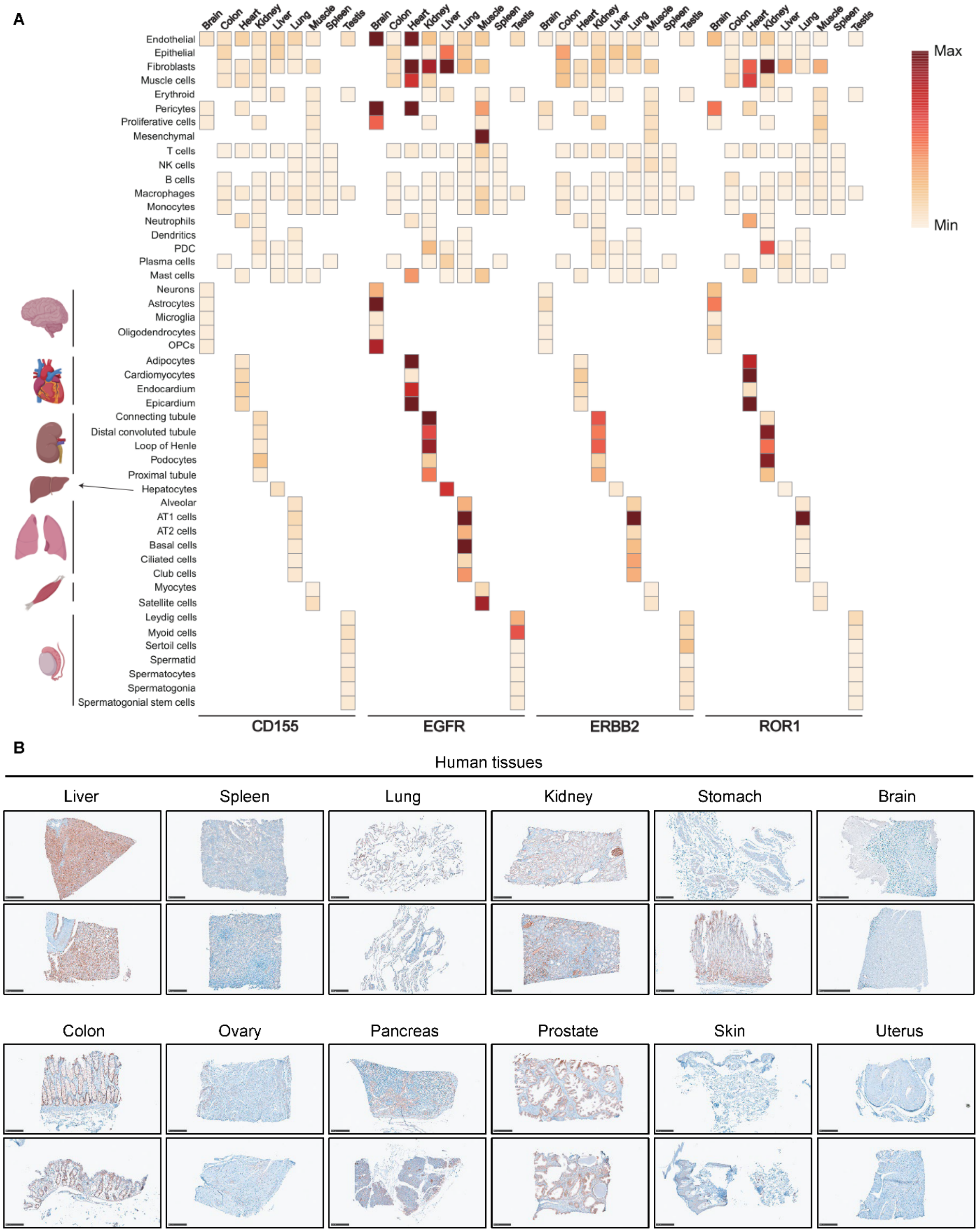
A
Brain
Colon
Heart
Kidney
Liver
Lung
Muscle
Spleen
Testis
Endothelial
Epithelial
Fibroblasts
Muscle cells
Erythroid
Pericytes
Proliferative cells
Mesenchymal
T cells
NK cells
B cells
Macrophages
Monocytes
Neutrophils
Dendritics
PDC
Plasma cells
Mast cells
Neurons
Astrocytes
Microglia
Oligodendrocytes
OPCs
Adipocytes
Cardiomyocytes
Endocardium
Epicardium
Connecting tubule
Distal convoluted tubule
Loop of Henle
Podocytes
Proximal tubule
Hepatocytes
Alveolar
AT1 cells
AT2 cells
Basal cells
Ciliated cells
Club cells
Myocytes
Satellite cells
Leydig cells
Myoid cells
Sertoil cells
Spermatid
Spermatocytes
Spermatogonia
Spermatogonial stem cells
CD155
EGFR
ERBB2
ROR1
Max
Min
B
Human tissues
Liver
Spleen
Lung
Kidney
Stomach
Brain
Colon
Ovary
Pancreas
Prostate
Skin
Uterus

Supplemental Figure 8 CD155 expression in normal human tissues. (**A**) Cross-Organ Off-Target Transcriptomic Atlas (COOTA) analysis assessed CD155 expression across various organs, using *EGFR*, *ERBB2* and *ROR1* genes as references. The single-cell transcriptomic atlas includes data from 49 scRNA-seq datasets spanning nine different organs, with each field representing the mean expression value per cluster (Supplemental Table 1). Blank fields indicate cell types that are not present in the study. (**B**) Immunohistochemical (IHC) analysis of CD155 expression in normal human tissues, including liver, spleen, lung, kidney, stomach, brain, colon, ovary, pancreas, prostate, skin and uterus (n = 2 individual donors). Scale bars represent 250 μm. Normal uterus previously shown in Supplemental Figure 4B is included again in this figure for comparison. Data represent the mean (**A**).

A

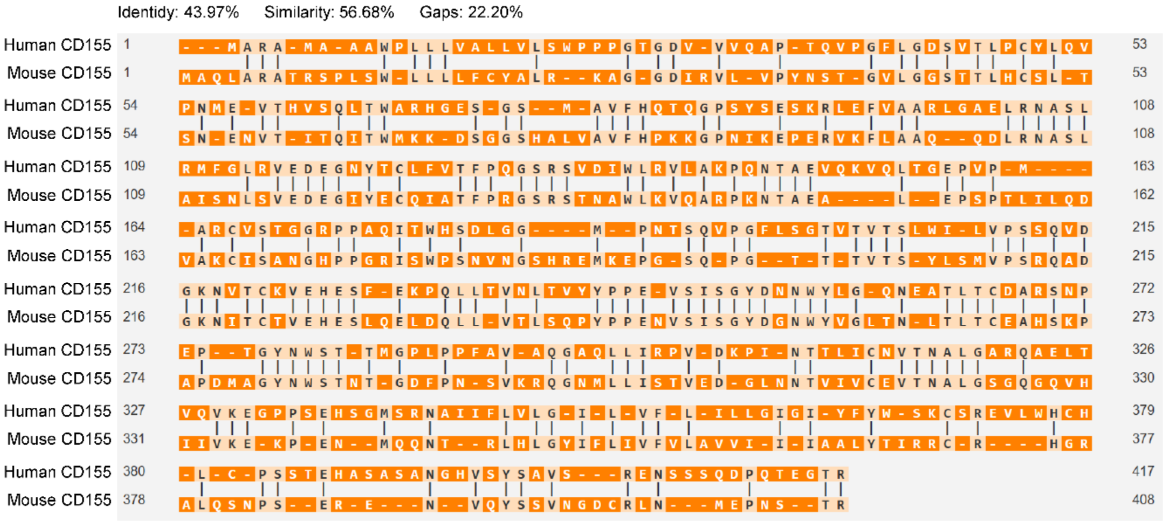

Supplemental Figure 9 Sequence alignment of human and mouse CD155 proteins. (**A**) A pairwise protein sequence alignment was performed between human CD155 and mouse CD155.

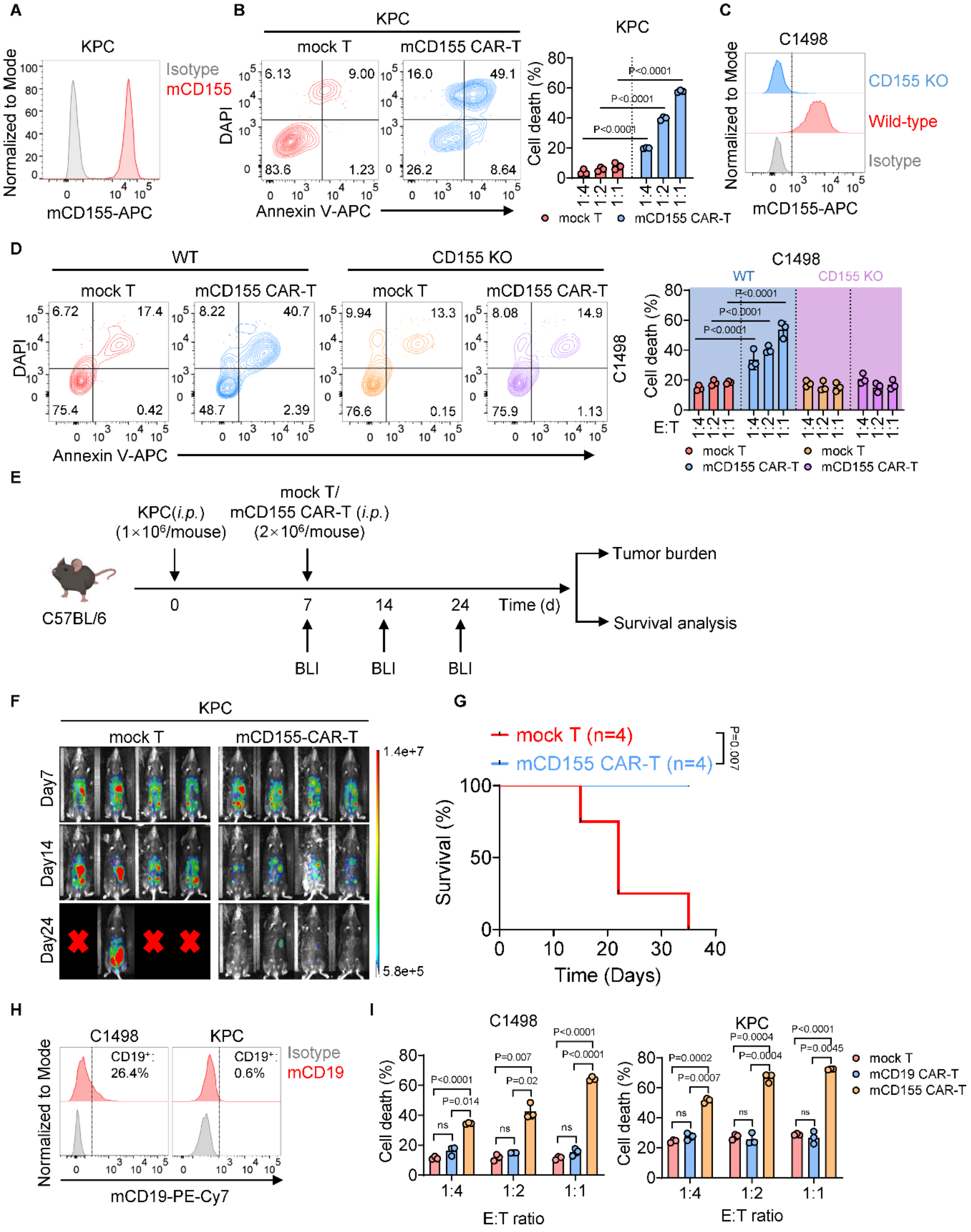
A
KPC
Isotype
mCD155
Normalized to Mode
mCD155-APC
B
KPC
mock T
mCD155 CAR-T
6.13
9.00
83.6
1.23
16.0
49.1
26.2
8.64
DAPI
Annexin V-APC
Cell death (%)
P<0.0001
mock T
mCD155 CAR-T
C
C1498
CD155 KO
Wild-type
Isotype
mCD155-APC
D
WT
CD155 KO
mock T
mCD155 CAR-T
6.72
17.4
75.4
0.42
8.22
40.7
48.7
2.39
9.94
13.3
76.6
0.15
8.08
14.9
75.9
1.13
Annexin V-APC
C1498
E:T
E
KPC(i.p.)
(1×10⁶/mouse)
mock T/
mCD155 CAR-T (i.p.)
(2×10⁶/mouse)
C57BL/6
Time (d)
BLI
Tumor burden
Survival analysis
F
KPC
mock T
mCD155-CAR-T
Day7
Day14
Day24
1.4e+7
5.8e+5
G
mock T (n=4)
mCD155 CAR-T (n=4)
P=0.007
Survival (%)
Time (Days)
H
C1498
KPC
CD19⁺: 26.4%
CD19⁺: 0.6%
Isotype
mCD19
Normalized to Mode
mCD19-PE-Cy7
I
C1498
KPC
Cell death (%)
E:T ratio
mock T
mCD19 CAR-T
mCD155 CAR-T

Supplemental Figure 10 Phenotypic and characterization of mCD155 CAR-T cells. (**A**) Representative histograms showing mCD155 expression in the murine pancreatic tumor cell line KPC. (**B**) Representative flow cytometry plots (left) and statistics (right) showing the percentage of KPC cell death co-cultured at indicated ratios with mock T or mCD155 CAR-T cells for 4 hours (n = 3 individual donors). (**C**) Representative histograms showing the expression of CD155 on wild-type and CD155-KO C1498 tumor cells. (**D**) Representative flow cytometry plots (left) and statistics (right) showing the percentage of wild-type or CD155-KO C1498 cell death co-cultured at indicated ratios with mock T or mCD155 CAR-T cells for 4 hours (n = 3 individual donors). (**E**) Diagram of the treatment scheme used for *in vivo* experiments. 1×10^6 KPC cells were i.p. injected into C57BL/6 mice, followed by an i.p. infusion of indicated number of mock T cells or mCD155 CAR-T cells (n = 4 mice per group). (**F** and **G**) Bioluminescence (BLI) images of tumor burden (**F**) and survival curves (**G**) in KPC tumor-bearing mice after different treatments. (**H**) Representative histograms showing the MFI of mCD19 and the percentage of mCD19 positive cells on C1498 and KPC cell lines. (**I**) Statistics showing the percentage of C1498 (left) and KPC (right) cell death co-cultured at indicated ratios with mCD19 CAR-T or mCD155 CAR-T cells for 4 hours (n = 3 individual donors). Data represent the mean ± S.D. and were analyzed by two-way ANOVA with repeated measures (**B**, **D** and **I**). For Kaplan-Meier survival curves, statistical significance was calculated with a log-rank test (**G**).

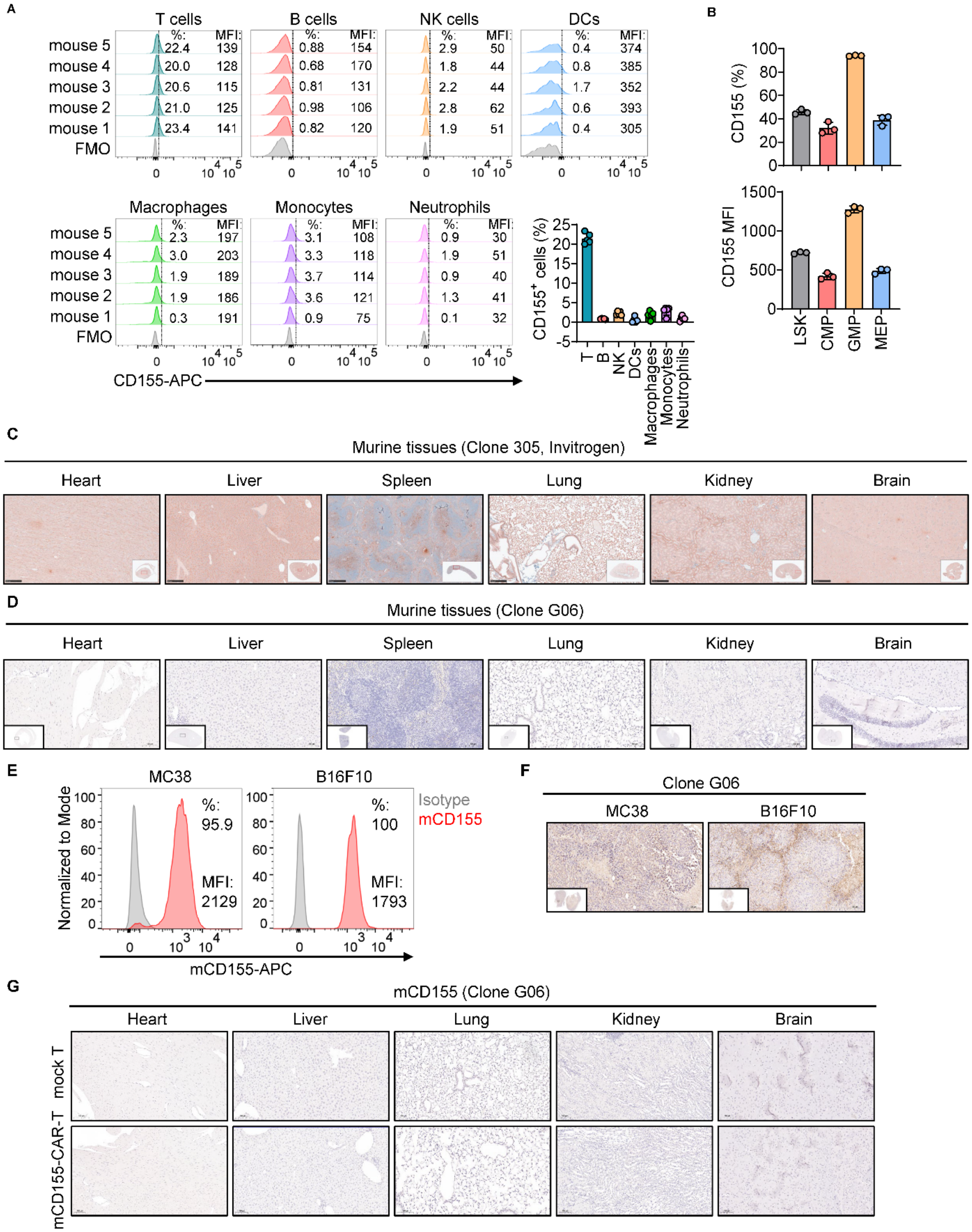
A
T cells
B cells
NK cells
DCs
mouse 5
mouse 4
mouse 3
mouse 2
mouse 1
FMO
Macrophages
Monocytes
Neutrophils
CD155-APC
CD155+ cells (%)
T
B
NK
DCs
Macrophages
Monocytes
Neutrophils
B
CD155 (%)
CD155 MFI
LSK
CMP
GMP
MEP
C
Murine tissues (Clone 305, Invitrogen)
Heart
Liver
Spleen
Lung
Kidney
Brain
D
Murine tissues (Clone G06)
E
MC38
B16F10
Normalized to Mode
Isotype
mCD155
mCD155-APC
F
Clone G06
G
mCD155 (Clone G06)
mock T
mCD155-CAR-T

Supplemental Figure 11 Murine CD155 expression profiles across hematopoietic and normal tissues. (**A**) Representative histograms and quantification of CD155 MFI and the percentage of $CD155^+$ cells across various mature immune cell subsets in mice (n = 5 individual mice). (**B**) Quantification of CD155 MFI and the percentage of $CD155^+$ cells across early hematopoietic progenitor populations in mice (n = 5 individual mice). Cell populations include: LSK (hematopoietic stem and multipotent progenitor cells), MPP (multipotent progenitor), CMP (common myeloid progenitor), GMP (granulocyte-macrophage progenitor), and MEP (megakaryocyte-erythroid progenitor). (**C**) Immunohistochemical (IHC) analysis of mCD155 expression in normal mouse tissues using the commercial anti-mCD155 antibody (clone 305, Invitrogen). Scale bars represent 250 µm. (**D**) Immunohistochemical (IHC) analysis of mCD155 expression in normal mouse tissues using the in-house G06 antibody. Scale bars represent 100 µm. (**E**) Flow cytometry histograms showing mCD155 expression on MC38 and B16F10 cell lines. (**F**) IHC analysis of mCD155 expression in MC38 and B16F10 tumors isolated from C57BL/6 mice, stained with the G06 antibody. Scale bars represent 100 µm. (**G**) IHC staining of mCD155 using the G06 antibody in the heart, liver, lung, kidney and brain from mice treated with mock T cells or mCD155 CAR-T cells. Scale bars represent 100 µm. Data represent the mean ± S.D. (**A** and **B**).

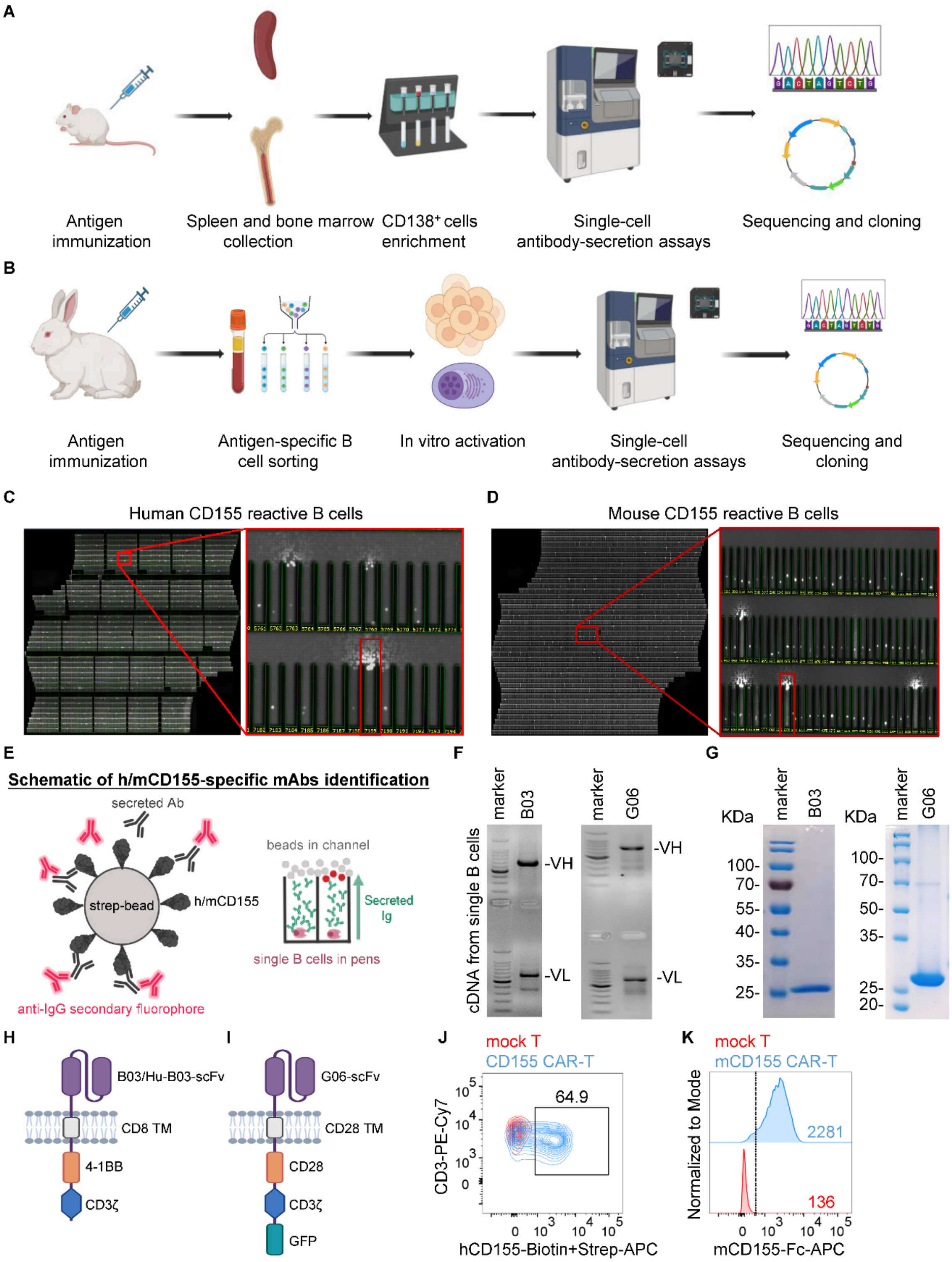
A
Antigen immunization
Spleen and bone marrow collection
CD138+ cells enrichment
Single-cell antibody-secretion assays
Sequencing and cloning
B
Antigen immunization
Antigen-specific B cell sorting
In vitro activation
Single-cell antibody-secretion assays
Sequencing and cloning
C
Human CD155 reactive B cells
D
Mouse CD155 reactive B cells
E
Schematic of h/mCD155-specific mAbs identification
secreted Ab
strep-bead
h/mCD155
anti-IgG secondary fluorophore
beads in channel
Secreted Ig
single B cells in pens
F
cDNA from single B cells
marker
B03
G06
-VH
-VL
G
KDa
marker
B03
G06
100-
70-
55-
40-
35-
25-
50-
20-
H
B03/Hu-B03-scFv
CD8 TM
4-1BB
CD3ζ
I
G06-scFv
CD28 TM
CD28
CD3ζ
GFP
J
mock T
CD155 CAR-T
64.9
CD3-PE-Cy7
hCD155-Biotin+Strep-APC
K
mock T
mCD155 CAR-T
2281
136
Normalized to Mode
mCD155-Fc-APC

Supplemental Figure 12 Generation and characterization of h/mCD155 CAR-T cells. (**A** and **B**) Workflow overview of anti-human CD155 (**A**) or anti-mouse (**B**) CD155 mAb development. (**C** and **D**) An example of an entire 11k chip is shown, with a zoomed-in view of a section to highlight the characteristic "blooms" over pens with cells secreting hCD155-reactive (**C**) or mCD155-reactive (**D**) antibodies. (**E**) Schematic of h/mCD155-specific mAbs identification. Left: Biotinylated antigen (dark grey) is coupled to a streptavidin-conjugated polystyrene bead (light grey). Antibodies (black Y-shaped) are secreted by single B cells loaded into individual NanoPens on the Berkeley Lights Beacon optofluidic instrument. Antibody binding to antigen was detected with a fluorescent anti-mouse IgG secondary antibody (red). Right: Schematic of fluorescing beads in the channel above a pen containing an individual B cell indicates antigen-specific reactivity. (**F**) PCR products of antibody heavy chains and light chains from individual B cells. (**G**) Reduced PAGE gel analysis showing the anti-hCD155 (B03) or anti-mCD155 (G06) scFvs migrating at approximately 25 kDa. (**H** and **I**) Schematic structure of the hCD155-CAR vector (**H**) or mCD155-CAR vector (**I**). (**J**) Representative flow cytometry plots showing the percentage of hCD155-CAR positive T cells on day 5 after transduction. (**K**) Representative histograms showing mCD155-CAR expression in T cells on day 5 after transduction.
